# Non-Invasive Assessment of Microvascular Invasion Risk in Hepatocellular Carcinoma Using Liquid Biopsy: Translational Insights and Clinical Implications

**DOI:** 10.3390/diagnostics16172686

**Published:** 2026-08-22

**Authors:** Dengyuan Xue, Xinyu Gao, Qixingmao Zhang, Hongxin Li, Mengli Chen, Xiuzhi Duan, Xuchu Wang, Pan Yu, Zhihua Tao, Xiaoxue Cheng

**Affiliations:** Department of Laboratory Medicine, Zhejiang University School of Medicine Second Affiliated Hospital, Hangzhou 310009, China; 18171125214@163.com (D.X.); 22418490@zju.edu.cn (X.G.); 15868757307@163.com (Q.Z.); lhx118753@163.com (H.L.); chenmengli74@163.com (M.C.); duanxiuzhi@zju.edu.cn (X.D.); wangxc@zju.edu.cn (X.W.); yupan107@zju.edu.cn (P.Y.)

**Keywords:** hepatocellular carcinoma, microvascular invasion, liquid biopsy, multi-omics integrated model, clinical management

## Abstract

Microvascular invasion (MVI) is a critical prognostic indicator for recurrence and survival in hepatocellular carcinoma (HCC); however, its accurate preoperative assessment remains clinically challenging. Postoperative histopathology is subject to sampling bias and time delays, while traditional imaging techniques lack the molecular specificity required to predict MVI. Liquid biopsy, through the analysis of circulating tumor DNA (ctDNA), circulating tumor cells (CTCs), circulating tumor RNA (ctRNA), and extracellular vesicles (EVs), provides a minimally invasive approach for capturing tumor-derived molecular and cellular signals associated with vascular invasion. This narrative review comprehensively summarizes the current evidence linking these four liquid biopsy analyte categories to MVI in HCC, evaluates their integration into multi-omics predictive models, including multi-marker, clinicopathological-integrated, and imaging-integrated strategies, and proposes an evidence-level framework that categorizes blood biomarkers according to the strength of their support for MVI prediction, distinguishing direct histopathological validation from indirect associations with aggressive tumor biology. Key challenges are critically examined, including the variable specificity of individual biomarkers for MVI, the lack of head-to-head comparative studies, the absence of standardized pre-analytical and analytical protocols, and the methodological limitations of current prediction models. As a narrative review, this work does not employ systematic review methodology, and the evidence synthesis should be interpreted accordingly. The review provides a framework for understanding how liquid biopsy-based MVI risk stratification may inform surgical and perioperative decision-making following prospective validation.

## 1. Introduction

Hepatocellular carcinoma (HCC), the predominant subtype of primary liver cancer, ranks as the sixth most commonly diagnosed malignancy and liver cancer remains the third leading cause of cancer-related deaths worldwide [1]. Its development is associated with various risk factors, such as the hepatitis B virus, alcohol consumption, and non-alcoholic fatty liver disease [2]. China is a region with a high prevalence of the hepatitis B virus; the number of newly diagnosed cases of HCC in the country accounts for nearly half of the global total [3,4], underscoring the immense disease burden faced by this population. Current treatment modalities for HCC include liver transplantation, surgical resection, and local ablation. Although these therapies prove effective for a subset of patients, they are limited by high rates of recurrence and metastasis, resulting in suboptimal long-term survival outcomes [5].

Vascular invasion is a prominent pathological feature of HCC and an important determinant of prognosis [6], closely associated with tumor stage and disease progression. From a histological perspective, it can be classified into macrovascular invasion and microvascular invasion (MVI). Macrovascular invasion typically indicates a relatively advanced stage of the disease, often precluding surgical intervention, and is associated with a poor prognosis [7]. In contrast, MVI may appear during the early stages of the disease and holds greater clinical diagnostic significance [8]. Although imaging techniques can detect major vascular invasion preoperatively, their limited resolution makes it difficult to reliably identify MVI. Currently, the diagnosis of MVI relies on postoperative tissue biopsy, an invasive procedure limited by the inherent variability of sample collection. The inherent shortcomings of these two detection methods have spurred extensive research into non-invasive alternatives.

Consequently, liquid biopsy is a highly promising non-invasive diagnostic tool. It shows considerable promise for the preoperative prediction of MVI, achieving this objective by capturing real-time tumor information. By analyzing tumor-derived components within bodily fluids including circulating tumor DNA (ctDNA), circulating tumor cells (CTCs), circulating tumor RNA (ctRNA), and extracellular vesicles (EVs), it provides dynamic molecular insights that are unattainable through traditional tissue biopsies or static imaging. Its capacity for longitudinal monitoring, the avoidance of sampling bias, and the reflection of tumor heterogeneity further underscores its clinical value in preoperative assessment. However, current evidence primarily demonstrates significant associations between liquid biopsy signals and MVI-related aggressive tumor features, whereas the specific causal and MVI-specific relationships remain largely speculative and lack direct prospective validation. To address this gap, this review adopts a structured evidence-classification approach (see Table 1), categorizing each biomarker according to the level of direct support for MVI prediction, ranging from histopathologically confirmed MVI association to mechanistic plausibility based on general tumor aggressiveness.

Previous reviews have addressed individual aspects of MVI assessment. Radiomics-based reviews have focused on imaging features [9], such as tumor margin irregularity, peritumoral enhancement, and texture analysis, for preoperative MVI prediction, without integrating liquid biopsy biomarkers. Liquid biopsy reviews in HCC have primarily addressed diagnosis, prognosis, and treatment monitoring rather than MVI-specific prediction [10], and have rarely compared different analyte classes within a unified framework. Reviews on multi-omics predictive modeling have discussed general integration strategies without using MVI as the central organizational endpoint [11].

**Table 1:** Evidence Classification of Blood Biomarkers for MVI Prediction.

Biomarker Type	Specific Marker	Level	Study Design	Cohort Size	Main Finding	Limitation	Ref.
ctDNA	ctDNA maximal VAF	A	Prospective	41 HCC patients (MVI+: 13; MVI−: 28)	ctDNA maximal VAF significantly higher in MVI+ patients (0.038 vs. 0.012, *p* = 0.0048). KEAP1, TP53, and NFKBIA mutations more frequent in MVI+ cases. ctDNA max VAF predicted MVI with AUC = 0.85 (95% CI: 0.693–0.998).	Moderate sample size (*N* = 41); single-center; requires external validation in larger independent cohorts.	[12]
ctDNA	DNA methylation haplotype blocks	A	Prospective	35 HCC patients (tissue + plasma; MVI+: 18; MVI−: 17) + 24 healthy controls	Tissue DNA methylation signature predicted MVI status with AUC = 85.9% (cross-validated) and was an independent predictor of MVI (OR = 47.51, *p* < 0.001). cfDNA methylation signature achieved AUC = 96.0% for HCC detection	Small sample size (*N* = 35); MVI prediction requires tissue biopsy, not liquid biopsy; cfDNA methylation signature could not distinguish MVI status; single-center.	[13]
ctDNA	Plasma methylated SEPT9	A	Retrospective	205 total (111 HCC [MVI+: 29; MVI−: 82] + 53 ARD + 41 healthy donors)	Plasma mSEPT9 independently predicted MVI (*p* = 0.002) and correlated with tumor number (*p* = 0.004), tumor size (*p* = 4.6 × 10^−5^), BCLC stage (*p* = 0.012). Combined mSEPT9 + PIVKA-II: AUC = 0.72 (sensitivity 62.1%, specificity 74.4%).	Retrospective design; single institution; MVI+ subgroup relatively small (*N* = 29); no power calculation reported.	[14]
ctDNA	ctDNA mutation burden (9-gene high-risk panel	C	Prospective	258 HCC patients undergoing curative resection	High-risk gene mutations predicted recurrence-free survival. Nomogram integrating ctDNA risk level and TNM stage achieved C-index = 0.76 (95% CI: 0.70–0.82). MVI was not a primary or secondary endpoint.	No MVI-specific endpoint; single-center; predominantly HBV-related HCC; systemic therapy subgroup was small (*N* = 29).	[15]
ctDNA	cfDNA fragmentation	B	Prospective/Retrospective	134 HCC patients (BCLC B/C; TKI: 83; ICI: 51)	High tumor fraction correlated with shorter PFS (3.0 vs. 10.6 months, *p* < 0.001) and OS (7.8 vs. 21.6 months, *p* = 0.02). High cfDNA fragmentation (>28%) associated with multinodularity (*p* = 0.02) and portal vein thrombosis (*p* = 0.003). No direct MVI histopathological validation performed.	Advanced-stage cohort (no early HCC); no independent validation cohort; MVI not assessed; single-center; preliminary.	[16]
CTC	Preoperative CTC count	A	Prospective	137 HCC patients (MVI+: 59; MVI−: 78) + 18 benign controls	Preoperative CTC count ≥ 5/5 mL was independent predictor of MVI (AUC = 0.856, sensitivity 91.4%, specificity 79.7%). Multi-parameter model (CTC + AFP + tumor diameter) achieved AUC = 0.900. CTC count correlated with MVI (*p* < 0.001), OS, and early recurrence risk.	Small sample size; short follow-up (median 25.5 months); single center; 8.2% sample failure rate with ISET method.	[17]
CTC	Preoperative EpCAM+ CTC count and MVI severity	A	Retrospective (Training + validation cohorts)	309 HCC patients (Training: 117; Validation: 192)	Preoperative CTC count positively correlated with MVI counts (r = 0.655, *p* < 0.001) and farthest MVI distance from tumor (r = 0.495, *p* < 0.001). CTC predicted MVI count > 5 with AUC = 0.744. For CTC+ patients, surgical margin > 1 cm independently protected against early recurrence (HR = 0.20–0.47).	Retrospective design; EpCAM-based CellSearch has limited sensitivity for EMT-CTCs; predominantly HBV-related HCC.	[18]
CTC	M-CTC count and M-CTC percentage	A	Prospective	127 HCC patients + 21 NMLD + 42 healthy controls	Total CTC count and M-CTC percent positively correlated with MVI (*p* = 0.0076 and *p* = 0.0231, respectively). M-CTC percent was strongest independent predictor of early recurrence (HR = 16.35, *p* < 0.0001). M-CTCs outperformed AFP in longitudinal surveillance (88.89% vs. 22.22%).	MVI was a secondary endpoint (primary: recurrence); only EMT-related CTC subtypes measured; relatively small sample for survival analysis; single center.	[19]
CTC	Vimentin+ CTCs	B	Prospective	80 patients (61 HCC + 11 NMLD + 8 healthy controls)	HCC-CTCs detected in 96.7% (59/61) of HCC patients. Vimentin+ CTCs discriminated early-stage (median: 0) from advanced-stage HCC (median: 6; AUC = 0.89, sensitivity 87.1%, specificity 90.0%). Significantly higher vimentin+ CTCs in patients with radiographic portal vein invasion (*p* < 0.001). Predicted OS (HR = 2.21) and recurrence after curative therapy (HR = 3.14).	MVI not directly assessed (radiographic portal vein invasion used as surrogate); single-center; median follow-up only 325 days; 41% of patients had prior therapy.	[20]
CTC	EMT markers in CTCs: Twist and vimentin co-expression	B	Retrospective	60 HCC patients + 10 benign + 10 healthy + 10 non-HCC cancers	Twist and vimentin were expressed in CTCs from 84.8% and 80.4% of CTC-positive patients, respectively. Co-expression of twist/vimentin in CTCs significantly correlated with portal vein tumor thrombus (PVTT), TNM stage, and tumor size. CTC positivity associated with higher recurrence/metastasis rate (88.0% vs. 16.7%).	Small sample size (*N* = 60); only 31 patients had ≥1-year follow-up; no multivariate analysis; single-center; MVI not specifically assessed	[21]
CTC	EMT-phenotype CTCs	C	Retrospective	33 HCC patients + 10 healthy volunteers	Epithelial CTCs correlated with tumor size (r = 0.456, *p* = 0.008); epithelial–mesenchymal-mixed CTCs (77.41% of all CTCs) correlated with tumor number (r = 0.421, *p* = 0.015); mesenchymal CTCs associated with metastasis (r = 0.375, *p* = 0.032).	Very small sample (*N* = 33); retrospective; no prognostic follow-up; no MVI assessment.	[22]
ctRNA (miRNA)	Serum miR-125b	A	Retrospective	108 HCC patients (MVI+: 32; MVI−: 76)	Serum miR-125b was independent predictor of MVI (*p* = 0.001). miR-125b alone: AUC = 76.97% (sensitivity 87.50%, specificity 51.32%). Combined with tumor size and AFP: AUC = 86.68% (sensitivity 84.38%, specificity 72.37%). Low miR-125b correlated with MVI and envelope invasion.	Retrospective design; single-center; limited sample size; specificity of miR-125b alone is modest (51.32%).	[23]
ctRNA (miRNA)	miR-497	D	Not applicable (mechanistic study)	36 paired HCC and adjacent non-tumor tissues + cell lines + nude mouse models	miR-497 significantly downregulated in HCC tissues and cell lines. Overexpression suppressed HCC angiogenesis (via VEGFA targeting) and metastasis (via AEG-1 targeting).	In vitro and animal models only; no clinical validation cohort; no liquid biopsy data; no direct MVI assessment	[24]
ctRNA (lncRNA)	Plasma lncRNAs: XLOC_014172, LOC149086	C	Case–control (multi-phase: screening, training, validation)	467 total (HCC + chronic hepatitis + controls); metastasis sub-analysis: 109 metastasis vs. 108 non-metastasis HCC	XLOC_014172 and LOC149086 significantly upregulated in metastatic HCC patients. Merged AUC for metastasis prediction: training set 0.900 (sensitivity 90%, specificity 90%); validation set 0.934 (sensitivity 91%, specificity 90%).	no direct MVI histopathological validation; mechanism not explored; preliminary study.	[25]
ctRNA (circRNA)	Serum circSETD3	A	Prospective	88 HCC patients (MVI+: 39; MVI−: 49) + 23 healthy controls	Low venous circSETD3 expression significantly associated with MVI (*p* = 0.023), gross vascular invasion (*p* = 0.002). circSETD3 was independent risk factor for MVI (OR = 0.458, *p* = 0.009). circSETD3 alone AUC = 0.637; combined with PIVKA-II: AUC = 0.736 (95% CI: 0.628–0.844).	Monocentric; relatively small sample (*N* = 88); follow-up < 1 year; venous circRNA levels not correlated with tissue levels.	[26]
ctRNA (circRNA)	Tissue circ_0008934	C	Retrospective	50 HCC patients (MVI+: 12; MVI−: 38) + paired non-tumor tissues	High circ_0008934 expression associated with MVI (*p* = 0.008), higher AFP (*p* < 0.001), larger tumor diameter (*p* = 0.012), and poorer prognosis (*p* = 0.007). Functions via miR-1305/TMTC3 axis to promote HCC proliferation, invasion, and migration in vitro and in vivo.	Tissue-based study; very small sample (*N* = 50); primary focus is mechanistic; MVI is one of several clinicopathological correlations; no AUC reported.	[27]
ctRNA (mRNA)	Serum S100P protein; Tissue S100P mRNA/protein	A	Retrospective (training) + Prospective (validation)	826 HCC patients (training + validation) + 89 healthy + 90 benign liver tumors; Tissue: 197 HCCs	S100P mRNA highly expressed in 92.9% (183/197) of HCC tissues, with further elevation in PVTT and MVI cases. Serum S100P discriminated HCC from benign liver tumors and predicted MVI status preoperatively (AUC = 0.744, sensitivity 62.4%, specificity 83.0%). S100P regulates CD44-mediated cell adhesion for PVTT/MVI formation.	7-point baseline sampling may yield false-negative MVI; single-center; lacked non-HCC malignant liver tumor cohort	[28]
EVs (mRNA)	Plasma exosomal PRPSAP1 mRNA	A	Prospective (discovery + validation cohorts)	131 HCC patients (Discovery: 37; Validation: 94; MVI+: 53; MVI−: 78)	Exosomal PRPSAP1 significantly higher in patients with >5 MVI foci vs. no MVI (0.147 vs. 0.070, *p* = 0.035). Other candidate mRNAs (RSAD2, HOXA2, CHMP4A) showed consistent trends but did not reach statistical significance between overall MVI+ and MVI− groups.	Overall MVI+ vs. MVI− comparison not statistically significant for any single marker; significant only in MVI > 5 subgroup; mechanism unexplored; single-center.	[29]
EVs (miRNA)	Serum exosomal miR-718	C	Retrospective	59 HCC patients undergoing living-donor LT + 6 screening patients + 58 resection patients (tissue validation)	HOXB8 identified as miR-718 target gene and associated with poor prognosis. Vascular invasion showed a trend toward association with low miR-718 but was NOT statistically significant (*p* = 0.13).	Small sample; could not confirm exosomal miRNA originated from HCC cells specifically; no MVI assessment.	[30]
EVs (miRNA panel)	Serum exosomal miRNAs: miR-18a, -221, -222, -224 (up); miR-101, -106b, -122, -195 (down)	C	Cross-sectional case–control	60 patients (CHB: 20; LC: 20; HCC: 20)	Serum exosomal miR-18a, miR-221, miR-222, and miR-224 significantly higher in HCC vs. CHB or LC (*p* < 0.05). miR-101, miR-106b, miR-122, and miR-195 lower in HCC vs. CHB. Authors explicitly stated that exosomal miRNA levels did NOT correlate with tumor characteristics including MVI, tumor stage, or differentiation.	Small sample size (*N* = 20 per group); no correlation with MVI or any tumor characteristics; single etiology (all HBV); HCC diagnosis study, not MVI prediction.	[31]
EVs (Protein)	Exosomal IGF2	D	Not applicable (mechanistic study)	HCC patient tissues and plasma + orthotopic mouse models	Fluid shear stress upregulated exosome secretion via Rab27a/Rab7. Exosomal IGF2 enriched in FSS-stimulated exosomes and promoted HCC migration/invasion via Ras/Raf/ERK signaling. In vivo, FSS-induced exosomes increased intrahepatic metastasis, blocked by IGF2 antagonist Xentuzumab. No MVI-specific clinical data.	Primarily mechanistic; clinical cohort size not specified; no MVI or vascular invasion endpoints; limited to cell migration/invasion and intrahepatic metastasis models.	[32]
EVs (Protein)	Exosomal CAP1	D	Not applicable (in vitro study)	No patient cohort (MHCC97-H and MHCC97-L HCC cell lines only)	Exosomes from high-metastatic MHCC97-H cells significantly increased migration and invasion of low-metastatic MHCC97-L cells. CAP1 was significantly enriched (4-fold) in MHCC97-H-derived exosomes. Proteomic profiling identified 129 exosomal proteins including CAP1, PPIA, KRT20, and ITIH4.	Cell line study only; no clinical patient samples; no in vivo validation; no MVI or vascular invasion data; purely mechanistic.	[33]

Abbreviations: AFP, alpha-fetoprotein; ARD, at-risk liver disease; BCLC, Barcelona Clinic Liver Cancer; CAP1, adenylate cyclase-associated protein 1; CHB, chronic hepatitis B; ddPCR, droplet digital polymerase chain reaction; EMT, epithelial–mesenchymal transition; FSS, fluid shear stress; GPC-3, glypican-3; GVI, gross vascular invasion; ICI, immune checkpoint inhibitor; IGF2, insulin-like growth factor 2; ISET, isolation by size of epithelial tumor cells; LC, liver cirrhosis; LT, liver transplantation; M-CTC, mesenchymal-phenotype circulating tumor cell; mSEPT9, methylated SEPT9; NGS, next-generation sequencing; NMLD, non-malignant liver disease; PFS, progression-free survival; PIVKA-II, protein induced by vitamin K absence or antagonist-II; PVTT, portal vein tumor thrombus; qRT-PCR, quantitative reverse transcription PCR; RFS, recurrence-free survival; TKI, tyrosine kinase inhibitor; VAF, variant allele frequency; VEGFA, vascular endothelial growth factor A.

The present review differs from existing work in three respects. First, it adopts an MVI-centric framework that organizes evidence around histologically confirmed MVI as the primary endpoint, rather than aggregating all biomarker associations related to HCC aggressiveness or recurrence. Second, it provides a comparative evaluation of four liquid biopsy analyte categories, namely ctDNA, CTCs, ctRNA, and EVs, with each category assessed according to a standardized evidence-level classification (Level A, direct pathological validation of MVI; Level B, indirect association with vascular invasion evidence; Level C, aggressive tumor phenotype evidence; and Level D, mechanistic plausibility). Third, it explicitly outlines the translational pathway from biomarker discovery to multi-omics model integration and ultimately to potential risk-adapted clinical management, while clearly distinguishing currently available evidence from future validation requirements. By maintaining a consistent focus on MVI as the unifying endpoint, this review provides a structured synthesis that complements existing general reviews of liquid biopsy applications in HCC. As a narrative review, the objective is not to provide an exhaustive catalog of all published biomarkers or integrated prediction models. Rather, representative studies were selected to illustrate the major biomarker categories, methodological approaches, and stages of clinical development currently reported in the field.

**Figure 1:**
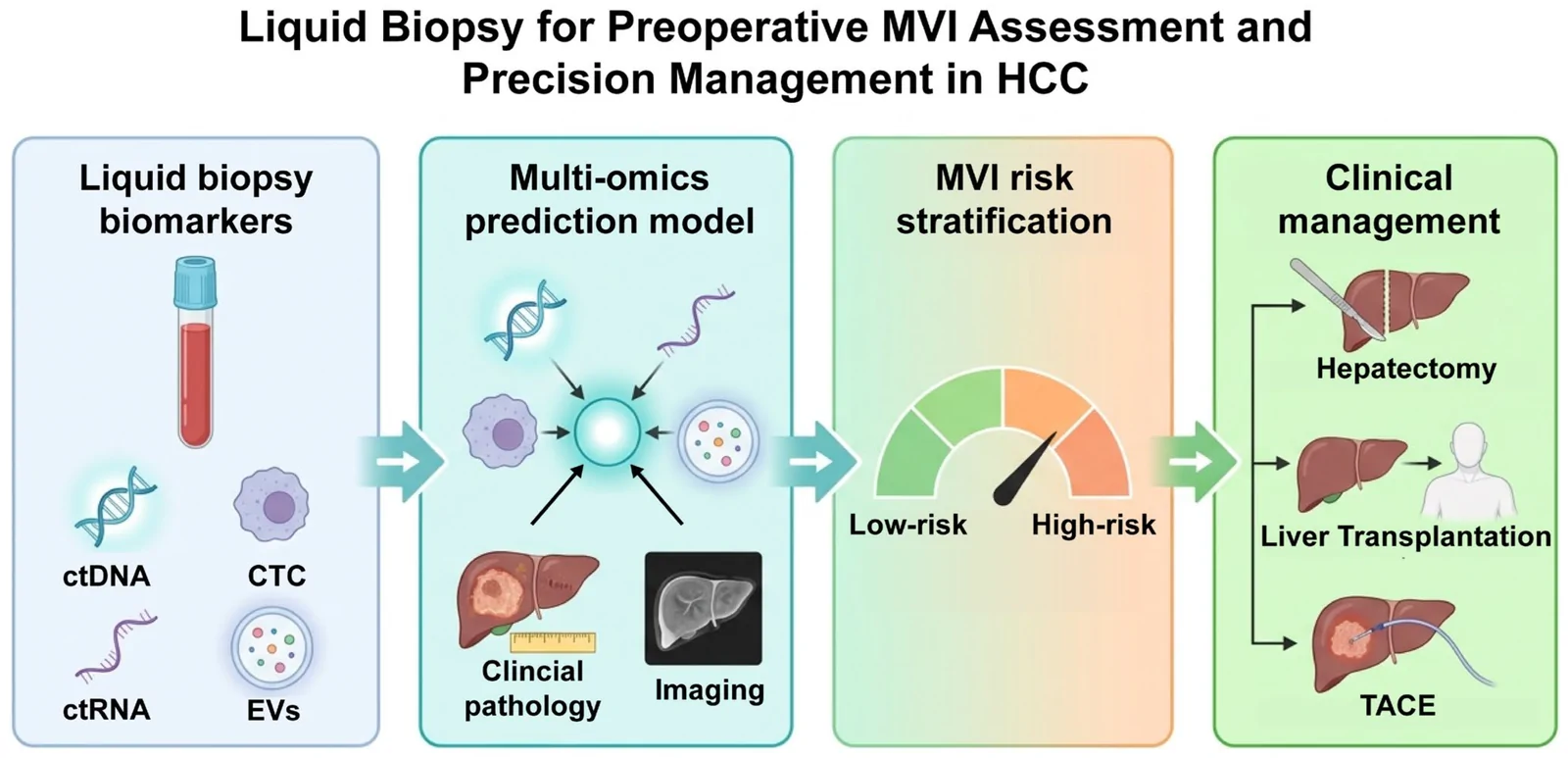
Schematic illustration of the liquid biopsy for preoperative MVI assessment and precision management in HCC. (1) Liquid biopsy biomarkers: peripheral blood is collected and analyzed for the four key circulating markers (ctDNA, CTCs, ctRNA, and EVs). (2) Multi-omics prediction model: the liquid biopsy signals are integrated with clinical pathological features and imaging data to construct a predictive model. (3) MVI risk stratification: patients are classified into low-risk or high-risk groups for MVI. (4) Clinical management: risk-adapted personalized management strategies are selected, including the selection of hepatectomy type, liver transplantation candidate selection, and adjuvant TACE administration.

This narrative review aims to provide a comprehensive overview of liquid biopsy biomarkers, including ctDNA, CTCs, ctRNA, and EVs, and their integration into multi-omics predictive models for the preoperative assessment of MVI in HCC. In addition, it explores the translational pathway from MVI risk stratification to individualized clinical management, with particular emphasis on the opportunities, current limitations, and future directions for clinical implementation (Figure 1).

## 2. Definition of MVI

**Figure 2:**
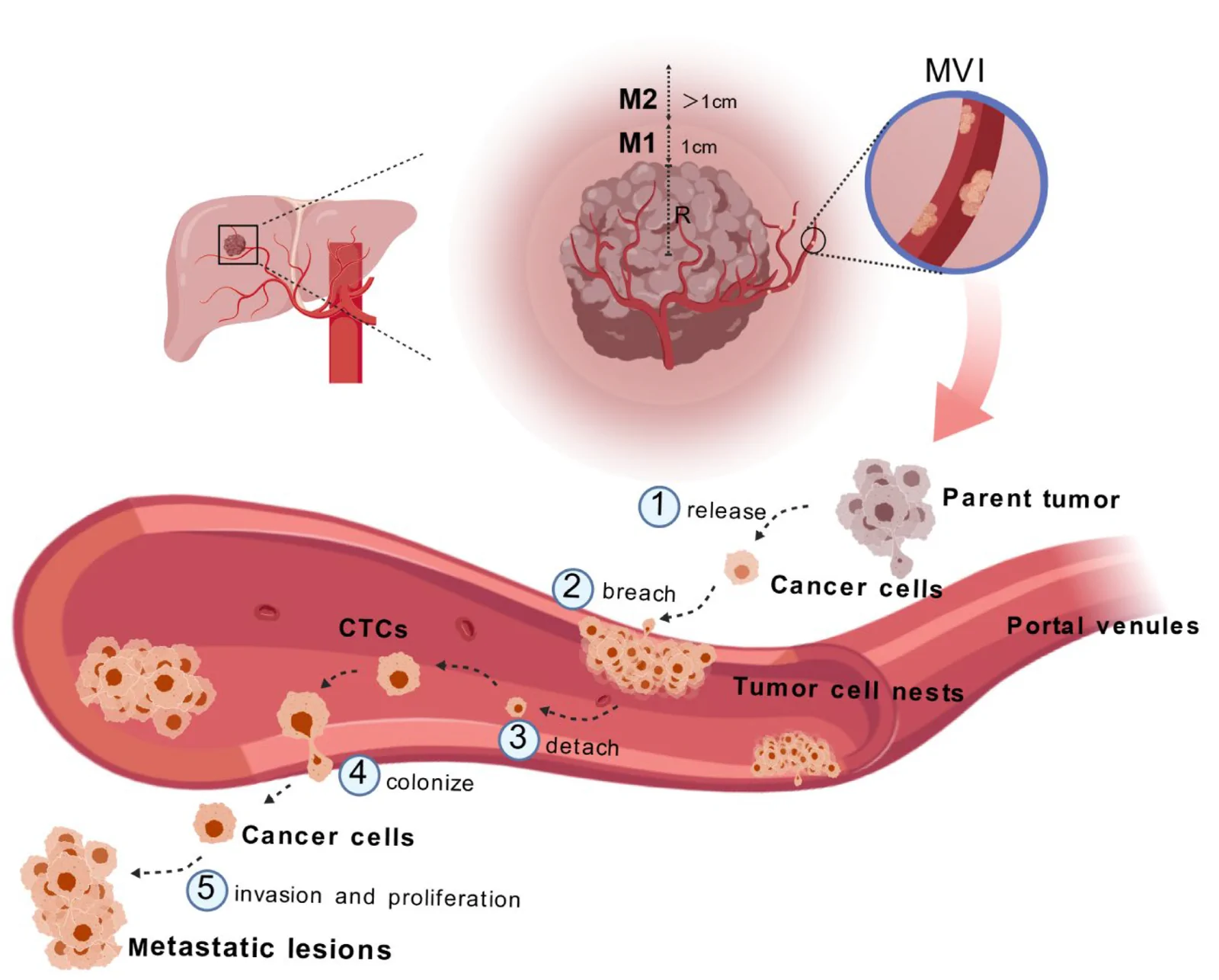
Schematic illustration of MVI development in HCC. (1) Invasive cancer cells detach from the parent tumor; (2) Cancer cells breach the vascular basement membrane and enter the vascular lumen, forming tumor cell nests; (3) Individual tumor cells dissociate from these nests and enter the bloodstream as CTCs; (4) CTCs travel through the circulation and adhere to suitable sites for colonization; (5) The colonized tumor cells proliferate and establish secondary metastatic lesions (Created with BioGDP.com [35]).

Histologically, MVI is defined as the presence of tumor cell clusters within vascular lumina lined by endothelial cells, typically occurring in the peritumoral regions of surgically resected specimens [34]. As a critical step in the process of tumor metastasis and dissemination, MVI begins with the detachment of invasive tumor cells from the primary site. These cells subsequently enter vascular channels and form tumor nests. These tumor nests may subsequently release CTCs into the bloodstream, thereby facilitating the formation of distant metastases (Figure 2).

Although MVI holds clear diagnostic significance in clinical practice, its diagnostic criteria remain subject to certain limitations due to variations in specimen sampling among pathologists, as well as inter-observer disagreement. These limitations in specimen sampling stem from early definitions regarding the portal or hepatic veins. Although Rodríguez-Peralvarez et al. later expanded the criteria to include arteries, arterioles, and lymphatics [36], this broader scope introduces additional variables into the selection of blood vessels. Inter-observer variability among pathologists further complicates the diagnostic process, as the interpretation of morphological features including tumor cluster size, the integrity of the endothelial lining, and proximity to the vascular lumen often varies significantly across different institutions [37]. To enhance diagnostic consistency and support clinical management, various MVI classification systems have been proposed. For instance, Roayaie et al. classified MVI into grades B1 through B3 based on the extent of muscular vascular invasion and its distance from the tumor [38]. Hwang et al. classified the severity of MVI (mild/severe) based on the number of invaded microvessels and tumor cells [39]. In China, Cong et al. established a risk stratification system and incorporated it into national guidelines. This system classifies MVI into the following categories: (1) M0, defined as no MVI detected; (2) M1 (low-risk), defined as the presence of ≤5 MVI foci within the hepatic tissue surrounding the tumor (≤1 cm from the tumor edge); (3) M2 (high-risk), defined as the presence of >5 MVI foci or any MVI located in the distant peritumoral liver tissue (>1 cm from the tumor edge). This comprehensive framework integrates international and domestic data regarding HCC, establishing a unified definition of MVI for the first time in China [40]. Although these systems can enhance reporting consistency and facilitate risk stratification, their clinical utility remains constrained by issues regarding sample representativeness and challenges in pathological interpretation. Consequently, further validation is required to substantiate their relevance in terms of prognosis and treatment.

## 3. MVI Detection

Accurate preoperative assessment of MVI in HCC is crucial for formulating personalized clinical management and conducting prognostic assessments [41]. As an indicator of tumor aggressiveness, MVI is closely associated with postoperative recurrence and metastatic potential. Consequently, the early and reliable identification of MVI is of great clinical importance, yet it remains a challenge. Currently, the assessment of MVI primarily relies on postoperative histopathology and preoperative imaging-based assessment. In recent years, liquid biopsy has increasingly been incorporated into the assessment framework for MVI in HCC, serving as a complementary, minimally invasive, and reproducible approach alongside tissue-based histopathological assessment and imaging. The biomarkers obtained through liquid biopsy primarily include CTCs, ctDNA, ctRNA, and EVs carrying tumor-derived nucleic acids and proteins. Collectively, these markers provide biological insights into tumor aggressiveness, thereby offering the potential to improve the preoperative prediction of MVI, as well as to facilitate risk stratification and long-term monitoring in the postoperative setting.

## 4. Tissue Biopsy and Imaging for MVI Detection

Regarding the assessment of MVI, traditional methods include invasive tissue biopsy and non-invasive imaging techniques. Tissue biopsy enables histopathological evaluation and remains the gold standard for the diagnosis of MVI. It allows for the direct visualization of the interactions between the tumor and blood vessels, and possesses high diagnostic specificity. However, its clinical application is subject to several major limitations. First, biopsy specimens are typically obtained during surgery and evaluated postoperatively, which limits their utility in preoperative clinical management. Moreover, the accuracy of MVI detection is strongly influenced by sampling strategies. The number and spatial distribution of sampling sites (NuSS) directly influence the detection rate. The traditional four-point sampling method primarily targets the tumor center and a limited surrounding area, which may fail to adequately cover the interface between the tumor and the liver, thereby leading to missed detections of MVI. To overcome these limitations, the present updated guidelines recommend a standardized 7-point sampling protocol that includes additional peritumoral regions and achieves a higher MVI detection rate [34]. Yet sampling errors and interpretive variability can still produce false-negative results [42]. Second, pathological examination is central to the tissue-based assessment of MVI, with H&E staining and immunohistochemical markers (e.g., CD34 and CK19) supporting MVI identification [37,43]. However, pathology is observer-dependent and largely morphology-based, providing static information that cannot capture dynamic molecular changes and has limited ability to predict recurrence patterns [44,45]. Finally, beyond these methodological limitations, tissue biopsy also encounters several practical clinical constraints, including invasiveness, limited patient acceptance, poor support for real-time or preoperative evaluation. Therefore, more flexible and non-invasive approaches are needed to achieve the prediction of MVI.

Non-invasive imaging modalities, including computed tomography (CT) and magnetic resonance imaging (MRI), are widely explored for MVI evaluation [46]. Prior studies have reported multiple tumor imaging features associated with MVI [47,48]. However, the reported correlations of specific features with MVI are often inconsistent, such as those related to tumor margin irregularity [49], reducing the clinical reliability of imaging-based assessment. Radiomics has emerged to address this gap by extracting high-dimensional imaging features for predictive modeling. Yet radiomics faces several challenges: (1) feature reproducibility remains poor due to inadequate standardization [50]; (2) few models have been developed for small HCCs, defined as tumors ≤3 cm in diameter [51]; (3) combined models from single-center studies show limited clinical applicability due to lacking external validation [52,53,54]. In addition, large-scale data processing and technical complexity hinder clinical translation.

Overall, traditional tissue biopsy and imaging techniques face limitations in providing real-time, preoperative, and biology-based assessments of MVI, as they rely primarily on static and indirect information. These shared limitations underscore the need for more flexible and actionable diagnostic strategies. In this context, liquid biopsy offers a promising alternative by enabling dynamic, preoperative, and molecularly informed evaluation of MVI.

## 5. Liquid Biopsy for MVI Detection

With the rise in minimally invasive diagnostics, liquid biopsy has become an important strategy for disease detection and monitoring. It refers to the analysis of body fluids, most commonly peripheral blood, to identify disease-related signals, particularly tumor-derived materials released into circulation during tumor growth, apoptosis, and necrosis [55]. The major analytes include ctDNA, CTCs, ctRNA, and EVs. All of which provide direct molecular evidence of tumor behavior. By providing accessible and repeatable molecular readouts, liquid biopsy complements tissue pathology and imaging, and is increasingly regarded as a key component of precision medicine for early detection, risk stratification, treatment selection, and longitudinal surveillance across oncology and other clinical fields.

As noted in the introduction, liquid biopsy offers unique advantages for MVI assessment, including non-invasiveness, real-time monitoring, and the capacity to capture tumor heterogeneity through serial blood sampling [56]. Accumulating evidence has demonstrated significant associations between MVI status and specific circulating biomarkers, particularly CTCs and ctDNA [57,58]. However, the strength of evidence supporting individual biomarkers varies considerably. To facilitate systematic evaluation, we have adopted an evidence-level classification framework: Level A (direct MVI histopathological validation), Level B (indirect vascular invasion evidence), Level C (aggressive tumor phenotype evidence), and Level D (mechanistic plausibility). In the following sections, we will comprehensively summarize the evidence linking each biomarker to MVI and provide an overview of the primary analytical platforms utilized for their detection, with a particular emphasis on their respective clinical utility.

## 6. Circulating Tumor DNA (ctDNA)

ctDNA is a tumor-derived fraction of cell-free DNA (cfDNA), consisting of short DNA fragments released into the bloodstream mainly through tumor-cell apoptosis and necrosis, typically ~100–150 bp in length [59]. By sampling tumor signals from multiple lesions and subclones, ctDNA can partially mitigate the spatial heterogeneity and sampling bias inherent to tissue biopsy [60]. Key ctDNA features, including somatic mutations, methylation signatures, and fragmentation patterns, have been investigated for their potential relevance to MVI and MVI-associated aggressive tumor phenotypes, although the strength of supporting evidence varies considerably. While some markers have been directly validated against histopathological MVI, many are supported primarily by indirect associations; the evidence level for individual ctDNA biomarkers is summarized in Table 1.

### 6.1. CtDNA Profiling

The mutational landscape captured by ctDNA reflects the aggressive biology of tumors. Higher ctDNA mutation burden has been associated with an increased risk of early postoperative recurrence (Level C) [15]. Direct evidence for MVI was provided by a prospective study of 41 patients, in which maximal ctDNA variant allele frequency (VAF) was significantly higher in MVI-positive than MVI-negative cases (0.038 versus 0.012; *p* = 0.0048), achieving an AUC of 0.85 (95% CI: 0.693–0.998) for preoperative MVI prediction (Level A) [12]. TP53, KEAP1, HIST1H3D, NFKBIA, PIK3CB and WRN mutations, together with enrichment of Rap1 and Ras signaling pathways, were more frequent in MVI-positive tumors [12]. These findings support the potential of ctDNA-based genomic profiling for non-invasive MVI risk assessment, although larger validation cohorts are still needed.

DNA methylation represents a stable epigenetic layer with potential utility for MVI prediction. A genome-wide methylation study of 35 HCC patients identified an eight-MHB (methylation haplotype block) tissue methylation signature that predicted MVI with a cross-validated AUC of 85.9% and remained an independent predictor of MVI (OR = 47.51; *p* < 0.001) (Level A) [13]. However, the corresponding cfDNA methylation signature, despite achieving an AUC of 96.0% for HCC detection, failed to discriminate MVI status, limiting its current applicability as a liquid biopsy marker [13]. In another retrospective cohort of 111 patients, plasma methylated SEPT9 (mSEPT9) was identified as an independent predictor of MVI (*p* = 0.002), with improved performance when combined with PIVKA-II (AUC = 0.72, sensitivity 62.1%, specificity 74.4%) (Level A) [14]. These data indicate that methylation signatures may contribute to both preoperative MVI prediction and prognostic assessment.

CtDNA fragmentation patterns. Beyond mutations and methylation, ctDNA fragmentation features, including fragment size, end motifs and nucleosome footprints, provide complementary information on tumor biology [61]. In a recent shallow whole-genome sequencing (sWGS) study of 134 patients with advanced HCC, elevated cfDNA fragmentation (>28%) was significantly associated with multinodularity (*p* = 0.02) and portal vein thrombosis (*p* = 0.003), representing indirect evidence of vascular invasion (Level B) [16]. Although direct associations with histopathologically confirmed MVI have not yet been established, the link between fragmentomic features and macrovascular invasion, together with their independent prognostic value, highlights ctDNA fragmentomics as a promising non-invasive biomarker that merits dedicated MVI-focused validation studies.

### 6.2. CtDNA Analysis Technologies

**Figure 3:**
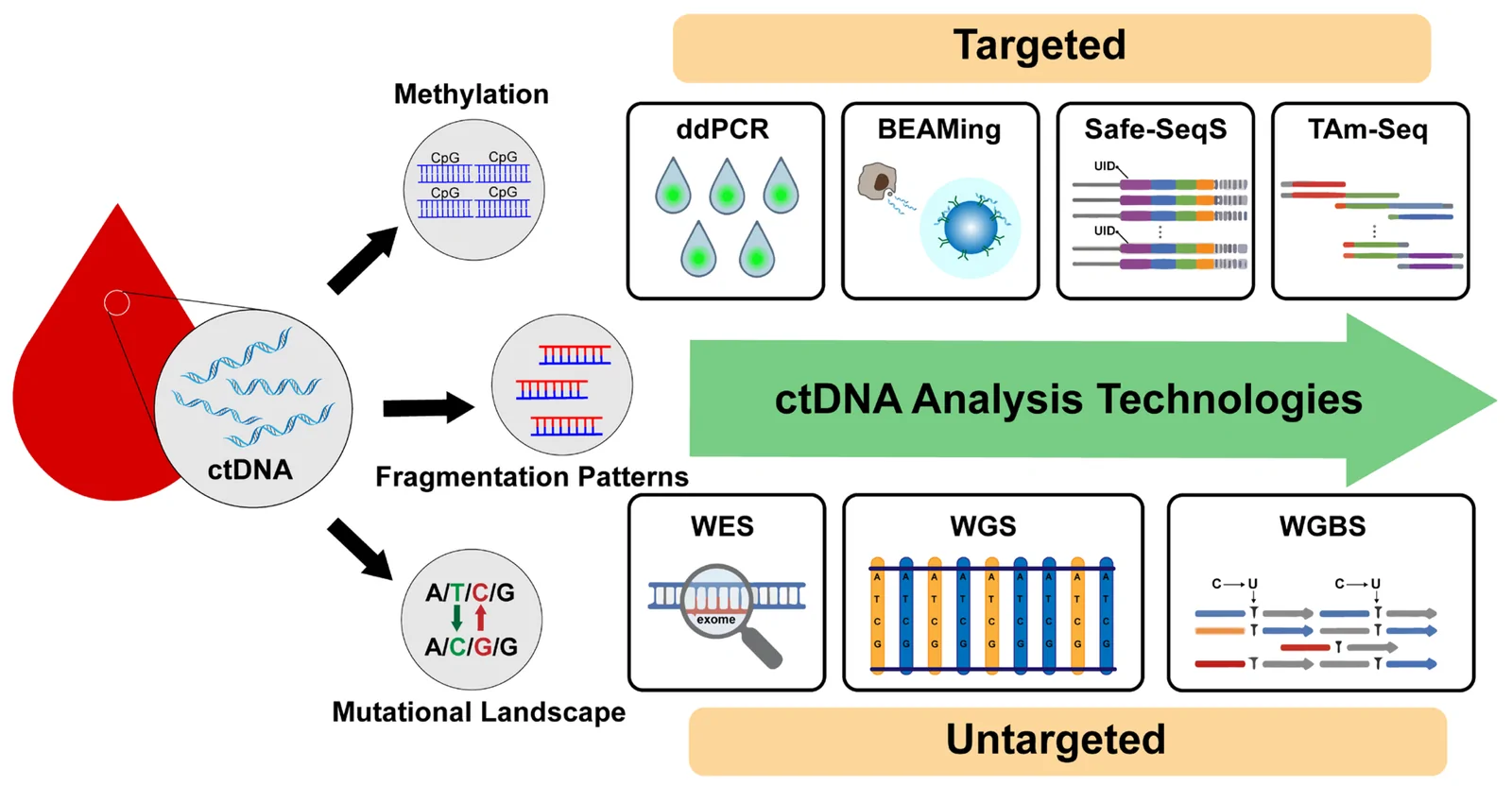
This schematic illustrates the main analytical features and analysis technologies of ctDNA. The analytical features include methylation patterns, fragmentation patterns, and mutational landscape. The analysis technologies are divided into targeted and untargeted methods. Targeted methods include droplet digital PCR (ddPCR), beads, emulsion, amplification, and magnetics (BEAMing), safe-sequencing system (Safe-SeqS), and tagged-amplicon sequencing (TAm-Seq). Untargeted methods comprise whole-exome sequencing (WES), whole-genome sequencing (WGS), and whole-genome bisulfite sequencing (WGBS).

Depending on the specific genomic regions of interest, ctDNA analysis techniques are generally categorized into targeted and non-targeted methods. The former screens for mutations within a pre-defined set of genes (e.g., single nucleotide variants), while the latter explores genomic aberrations (e.g., WGS). Targeted methods include droplet digital PCR (ddPCR) [62], beads, emulsion, amplification, and magnetics (BEAMing) [63], safe-sequencing system (Safe-SeqS) [64], cancer personalized profiling by deep sequencing (CAPP-Seq), and tagged amplicon deep sequencing (TAm-Seq) [65]. These technologies possess extremely high sensitivity (with a detection limit for mutant allele frequencies as low as 0.01%) and high specificity for known mutations; they are, therefore, highly suitable for monitoring treatment responses in MVI patients [66]. However, their reliance on prior knowledge of mutations limits the discovery of novel biomarkers [67]. Untargeted methods primarily involve next-generation sequencing (NGS) which provide comprehensive genomic profiling, such as whole exome sequencing (WES) and WGS [68,69], but face challenges in sensitivity and cost-effectiveness for routine MVI screening [70]. Based on enrichment strategies, NGS-based techniques are further classified as targeted amplification sequencing (TAS) and targeted capture sequencing (TCS). These technologies enable the simultaneous assessment of multiple molecular features and therefore hold considerable potential for preoperative MVI evaluation [71]. However, although some studies have reported direct associations with histopathologically confirmed MVI, much of the current evidence remains derived from correlations with aggressive tumor characteristics and clinical outcomes, highlighting the need for further prospective validation. In addition, while NGS facilitates the noninvasive characterization of tumor heterogeneity, persistent challenges remain in assay standardization, data interpretation, and the discrimination of authentic tumor-derived signals from sequencing artifacts [72]. Specific detection methods can be found in Table 2 [73]. Classifications of current detection technologies are presented in Figure 3.

### 6.3. Confounding Factors in Liquid Biopsy Interpretation

The interpretation of circulating biomarkers for MVI prediction must account for several biological and clinical factors that can influence liquid biopsy measurements independently of MVI status. Liver cirrhosis, present in the majority of HCC patients and particularly prevalent in Asian populations, elevates background cfDNA levels through chronic hepatocyte turnover and may alter DNA methylation patterns independently of tumor biology [92]. Active viral hepatitis, particularly during alanine aminotransferase (ALT) flares, can cause non-specific cfDNA release from inflamed but non-malignant hepatocytes, potentially confounding ctDNA-based MVI prediction [93]. Prior locoregional therapy, including transarterial chemoembolization (TACE) and radiofrequency ablation (RFA), induces tumor necrosis that transiently elevates ctDNA levels, complicating the interpretation of post-treatment liquid biopsy measurements [62]. Systemic therapies, including tyrosine kinase inhibitors and immune checkpoint inhibitors, can modulate CTC counts and phenotypes through both anti-tumor effects and off-target hematopoietic alterations [94]. Furthermore, background liver injury, steatohepatitis, and systemic inflammation may alter EV composition and ctRNA profiles independently of MVI [95]. Hemolysis during blood collection releases erythrocyte-derived miRNAs that can dominate small RNA sequencing libraries and mask tumor-derived signals [96]. These confounders underscore the critical importance of well-characterized control populations with documented liver disease etiology, severity, treatment history, and inflammatory status when evaluating liquid biopsy-based MVI prediction. Standardized reporting of these clinical variables should be adopted in future studies to enable meaningful cross-study comparisons.

## 7. Circulating Tumor Cells (CTCs)

CTCs are cancer cells that detach from primary solid tumors, intravasate into the bloodstream or lymphatic system, and may eventually form metastases in distant organs. In HCC, both the presence and biological characteristics of CTCs have important clinical implications and have been associated with tumor progression, metastatic potential, and MVI [18,97]. Given the marked heterogeneity of circulating tumor cells (CTCs) in both biological characteristics and abundance, CTC-based analyses are generally approached from two complementary perspectives: phenotypic profiling and quantitative assessment. Based on the expression of epithelial and mesenchymal markers, CTCs can be broadly classified into epithelial, mesenchymal, and hybrid epithelial–mesenchymal transition (EMT) phenotypes, reflecting varying degrees of cellular plasticity associated with tumor dissemination and metastatic potential. Among circulating biomarkers, CTCs, particularly mesenchymal and hybrid EMT phenotypes, provide relatively direct cellular evidence of vascular dissemination relevant to MVI. However, the strength of supporting evidence varies considerably across individual CTC biomarkers and study designs, as summarized in Table 1.

As discussed in ctDNA, confounding clinical factors, including liver disease etiology, prior locoregional therapy, and systemic treatment, should be carefully considered when interpreting CTC data for MVI prediction.

### 7.1. CTCs Phenotypic Profiling

Epithelial-phenotype CTCs (E-CTCs), characterized by the expression of epithelial markers such as EpCAM and cytokeratins, are generally considered to indicate the shedding of tumor cells from the primary lesion site. Clinical studies have confirmed a positive correlation between E-CTCs and tumor burden (r = 0.456, *p* = 0.008) [22]. Elevated E-CTC levels are associated with adverse treatment outcomes in HCC patients [98]. However, direct evidence demonstrating that E-CTCs can serve as specific preoperative biomarkers for MVI remains limited (Level C).

Mesenchymal-phenotype CTCs (M-CTCs), representing tumor cells that have undergone EMT and therefore exhibit enhanced motility and invasiveness [99], have demonstrated predictive value for postoperative recurrence [19] and correlate with metastatic risk and adverse clinicopathological features, including elevated AFP levels, advanced tumor stage, and vascular invasion [22,100]. In a prospective study of 127 HCC patients, M-CTC percentage was positively correlated with MVI status (*p* = 0.0076) (Level A) [19], suggesting that the mesenchymal CTC phenotype may reflect aggressive tumor behavior relevant to MVI-associated invasion patterns.

EMT-phenotype CTCs (EMT-CTCs), characterized by the downregulation of epithelial markers (e.g., EpCAM/CK) and the upregulation of mesenchymal markers (e.g., vimentin), represent hybrid subpopulations capturing dynamic intermediate states during tumor progression [101]. Higher detection rates of vimentin-positive CTCs have been observed in patients with portal vein invasion (Level B) [20], and twist/vimentin co-expression has been associated with portal vein tumor thrombus, TNM stage, and tumor size (Level B) [21]. Although these findings support a potential relationship between EMT-related CTC phenotypes and vascular invasive behavior, direct evidence linking specific CTC subtypes to MVI remains insufficient and warrants further prospective validation.

### 7.2. Quantitative Assessment of CTC

Quantitative CTC enumeration provides one of the strongest evidence bases for preoperative MVI prediction. In a prospective study of 137 HCC patients, a preoperative CTC count ≥ 5/5 mL was an independent predictor of MVI (AUC = 0.856, sensitivity 91.4%, specificity 79.7%), outperforming both tumor diameter (AUC = 0.604) and AFP (AUC = 0.636); the multi-parameter combination (CTC + AFP + tumor diameter) achieved an AUC of 0.900 (Level A) [17]. A separate retrospective study of 309 HCC patients further demonstrated that preoperative CTC count positively correlated with MVI counts (r = 0.655; *p* < 0.001) and the farthest MVI distance from the tumor margin (r = 0.495; *p* < 0.001), and that CTC-positive patients specifically benefited from a surgical margin > 1 cm for protection against early recurrence (HR = 0.20–0.47) (Level A) [18]. These studies collectively establish quantitative CTC assessment as one of the most clinically advanced liquid biopsy strategies for preoperative MVI evaluation, although the reliance on EpCAM-based capture in one study may underestimate the contribution of EMT-CTCs [18].

### 7.3. CTCs Analysis Technologies

**Figure 4:**
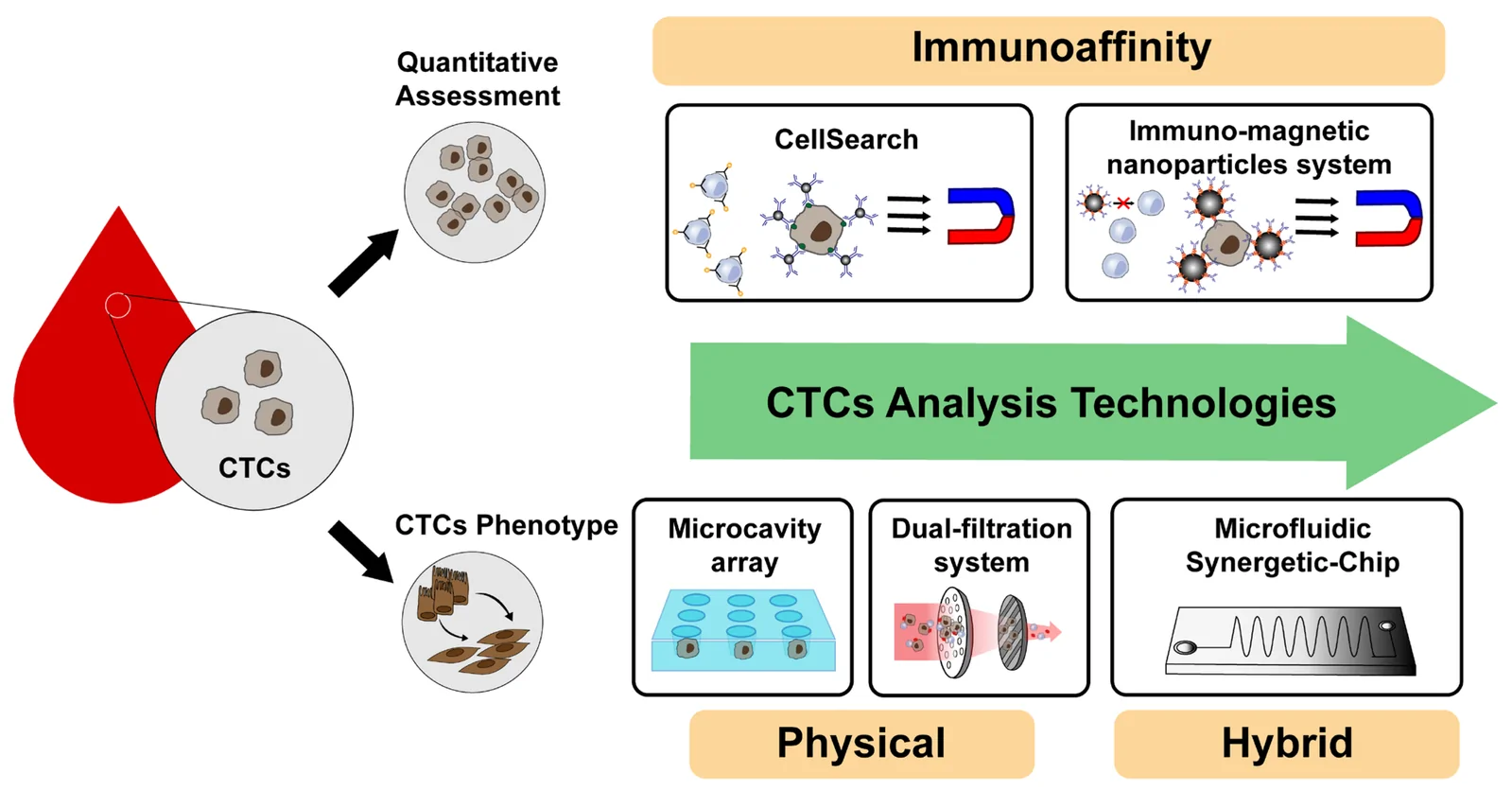
This schematic illustrates the main analytical features and analysis technologies of CTCs. The analytical features include CTC phenotypic characterization and quantitative assessment. The analysis technologies are divided into immunoaffinity, physical, and hybrid methods. Immunoaffinity methods include the CellSearch system and immuno-magnetic nanoparticles system (IMNs). Physical methods include the microcavity array and the dual-filtration system. Hybrid technologies include the microfluidic synergetic-chip.

According to their isolation principles, current CTC analysis technologies are generally classified into immunoaffinity, physical, and hybrid methods. Immunoaffinity methods primarily rely on EpCAM-based antigen–antibody interactions, as exemplified by the FDA-approved CellSearch system [74]. The primary advantage of this method lies in its high specificity for epithelial CTCs and the reliability of its clinical validation. However, a major limitation is its inability to capture EMT-CTCs due to EpCAM downregulation during EMT, severely restricts its utility in MVI prediction where mesenchymal and hybrid CTCs phenotypes may be of particular importance [102]. Physical techniques (including microfiltration, and density gradient centrifugation) offer the advantage of marker-independent CTCs isolation, potentially capturing the full spectrum of CTCs heterogeneity [103], Nevertheless, these methods suffer from lower purity due to leukocyte contamination and may compromise cell viability through mechanical stress [104]. To enhance detection efficiency, a hybrid strategy combining physical enrichment with immunoaffinity validation (e.g., molecular characterization) represents the most promising method for detecting MVI [105], thereby enabling the functional analysis of captured EMT-CTCs. However, barriers related to standardization, as well as technical complexities, have hindered clinical translation, particularly in the context of CTC subpopulations associated with rare MVI [106]. Specific detection methods can be found in Table 2 [75,76,77,78]. Classifications of current CTCs detection technologies are presented in Figure 4.

## 8. Circulating Tumor RNA (ctRNA) and Extracellular Vesicles (EVs)

The cell-free RNA (cfRNA) refers to RNA released into circulation by cells through death, necrosis or active secretion [107]. The ctRNA, a tumor-derived subset of cfRNA, comprises coding RNAs (e.g., mRNA fragments) and non-coding RNAs (ncRNAs). The latter encompasses microRNAs (miRNAs), long non-coding RNAs (lncRNAs), and circular RNAs (circRNAs). The ctRNA contributes to cancer progression by regulating key processes, including cell proliferation, migration, invasion, and apoptosis. Its circulatory stability, tissue-specific expression, and capacity to distinguish between malignant and non-malignant lesions make it an attractive class of biomarker for liquid biopsy [108].

EVs including exosomes, microvesicles, and apoptotic bodies are lipid bilayer-enclosed vesicles with a diameter ranging from approximately 50 nm to more than 1000 nm that carry nucleic acids, proteins, and lipids [109]. Through systemic dissemination, EVs mediate intercellular communication and contribute to tumor progression and metastasis [110]. Their ability to encapsulate both nucleic acids and proteins from parent tumor cells makes them particularly valuable for liquid biopsy applications. In HCC, tumor-derived EVs can deliver oncogenic RNA to recipient cells, promoting tumor growth and invasion [111]. While EV-associated proteins also reflect molecular features relevant to metastatic behavior [112]. Collectively, these characteristics underpin the growing interest in both EV-carried RNA (including mRNA and ncRNA) and EV proteomic signatures as potential biomarkers for MVI. As with other emerging circulating analytes, the evidence supporting the relevance of ctRNA and EV biomarkers to MVI spans a spectrum from direct pathological correlation to indirect clinical associations and mechanistic plausibility; this evidence gradient is systematically summarized in Table 1.

As discussed in ctDNA, the interpretation of ctRNA and EV biomarkers for MVI prediction must account for confounding factors including liver disease etiology, treatment history, and pre-analytical variables such as hemolysis, which can substantially alter circulating RNA profiles.

### 8.1. CtRNA Profiling

miRNAs are short ncRNAs characterized by high stability and resistance to degradation, making them attractive candidates as liquid biopsy biomarkers [113]. Among the strongest Level A evidence for ctRNA-based MVI prediction, serum miR-125b was identified as an independent predictor of MVI in a cohort of 108 HCC patients; when combined with tumor size and AFP, the predictive model achieved an AUC of 0.867 (sensitivity 72.37%, specificity 84.38%) [23]. Mechanistically, angiogenesis, known to correlate closely with MVI [114], may be facilitated by decreased miR-497, which derepresses VEGFA and AEG-1 to promote vascular endothelial signaling in HCC (Level D) [24].

lncRNAs, typically longer than 200 nucleotides, possess stable secondary structures that support their detectability in circulating fluids [115]. Plasma lncRNAs XLOC_014172 and LOC149086 were significant upregulated in metastatic HCC patients, yielding AUC of 0.900 (training set) and 0.934 (validation set) for MVI prediction; however, the endpoint was broadly defined as metastasis rather than MVI-specific, placing this evidence at Level C [25].

circRNAs are covalently closed RNA molecules that regulate cancer progression by modulating signaling pathways and functioning as oncogenes or tumor suppressors [116]. Prior research have studied that serum circSETD3 was identified as an independent risk factor for MVI (OR = 0.458; *p* = 0.009), with an AUC of 0.637 alone and 0.736 (95% CI: 0.628–0.844) when combined with PIVKA-II (Level A) [26]. Tissue-based studies have also demonstrated that elevated circ_0008934 expression is significantly associated with MVI (*p* = 0.008), functioning through the miR-1305/TMTC3 axis to promote proliferation and metastasis (Level C, tissue-based) [27].

mRNAs, especially when integrated with proteomic analysis, enhance the accuracy of cancer detection and characterization [117]. Serum S100P mRNA, integrating transcriptomic and proteomic analysis, was highly expressed in 92.9% (183/197) of HCC tissues, with further elevation in PVTT and MVI cases; serum S100P protein predicted MVI preoperatively with an AUC of 0.744 (sensitivity 62.4%, specificity 83.0%) across a large combined retrospective and prospective cohort of 826 patients (Level A) [28].

### 8.2. EV Profiling

EV-encapsulated RNAs are protected by the vesicular lipid bilayer, conferring enhanced stability in blood and enabling sensitive detection while potentially capturing tumor heterogeneity [118,119]. A recent prospective study of 131 HCC patients demonstrated that plasma exosomal PRPSAP1 levels were significantly higher in patients with >5 MVI foci compared with those without MVI (0.147 versus 0.070; *p* = 0.035), providing direct evidence linking exosomal mRNA to MVI status (Level A) [29]. However, the overall MVI-positive versus MVI-negative comparison did not reach statistical significance for any single exosomal mRNA, indicating that the discriminatory power of this approach is currently restricted to high-MVI-burden disease. Several studies have reported dysregulated exosomal miRNA profiles in HCC, including altered expression of miR-21-5p, miR-10b-5p, miR-221-3p, and miR-718. Although these molecules are associated with tumor aggressiveness and recurrence risk, direct evidence verifying their relationship with MVI is lacking (Level C) [30,31,120].

Exosomal proteomics, reflecting the molecular composition and metastatic propensity of the parent tumor, also provides candidate biomarkers in HCC. Adenylate cyclase–associated protein 1 (CAP1), enriched in exosomes from highly metastatic HCC cell lines, correlates with metastatic propensity, and insulin-like growth factor 2 (IGF2) enrichment in biomechanical stress-induced exosomes promotes migratory signaling via the Ras/Raf/ERK pathway; both currently remain at the mechanistic plausibility level (Level D) [32,33]. Collectively, EV-based biomarkers for MVI are at an earlier stage of clinical development compared with ctDNA- and CTC-based approaches, and most findings should be regarded as preliminary associations requiring confirmation in larger, MVI-focused prospective studies.

### 8.3. Analysis Technologies for ctRNA and EVs

In the analysis of ctRNA, analytical strategies are primarily designed according to the readout format and assay objective, and can be broadly classified into amplification-based quantitative techniques and capture-based sequencing techniques. Amplification-based methods include reverse-transcription droplet digital PCR (RT-ddPCR), which enables ultra-sensitive detection of low-abundance transcripts [79,80], and quantitative reverse transcription PCR (qRT-PCR), a rapid and cost-effective option for routine measurement [81]. In contrast, capture-based platforms such as targeted RNA sequencing (Target-RNAseq) provide multiplexed analysis of predefined transcript panels [82], supporting downstream identification of expression signatures relevant to MVI.

In the analysis of EVs, methodological workflows typically involve multiple sequential steps. Based on the key analytical objectives, EV analysis encompasses isolation, characterization, and molecular profiling. Isolation techniques include ultracentrifugation (accessible but time-consuming/impractical) [83], size-exclusion chromatography (SEC; relatively pure plasma EVs are rapid but yield-limited) [84], affinity capture (specific but potentially biased) [85], or emerging methods like microfluidics enhancing efficiency [86]. Characterization uses electron microscopy [87], nanoparticle tracking analysis (NTA) [88] and flow cytometry [89]. Molecular profiling shows significant potential, with EV proteomics aiding cancer detection [90,121] and small RNA sequencing (Small RNA-seq) revealing functional EV RNA cargo [91].

**Figure 5:**
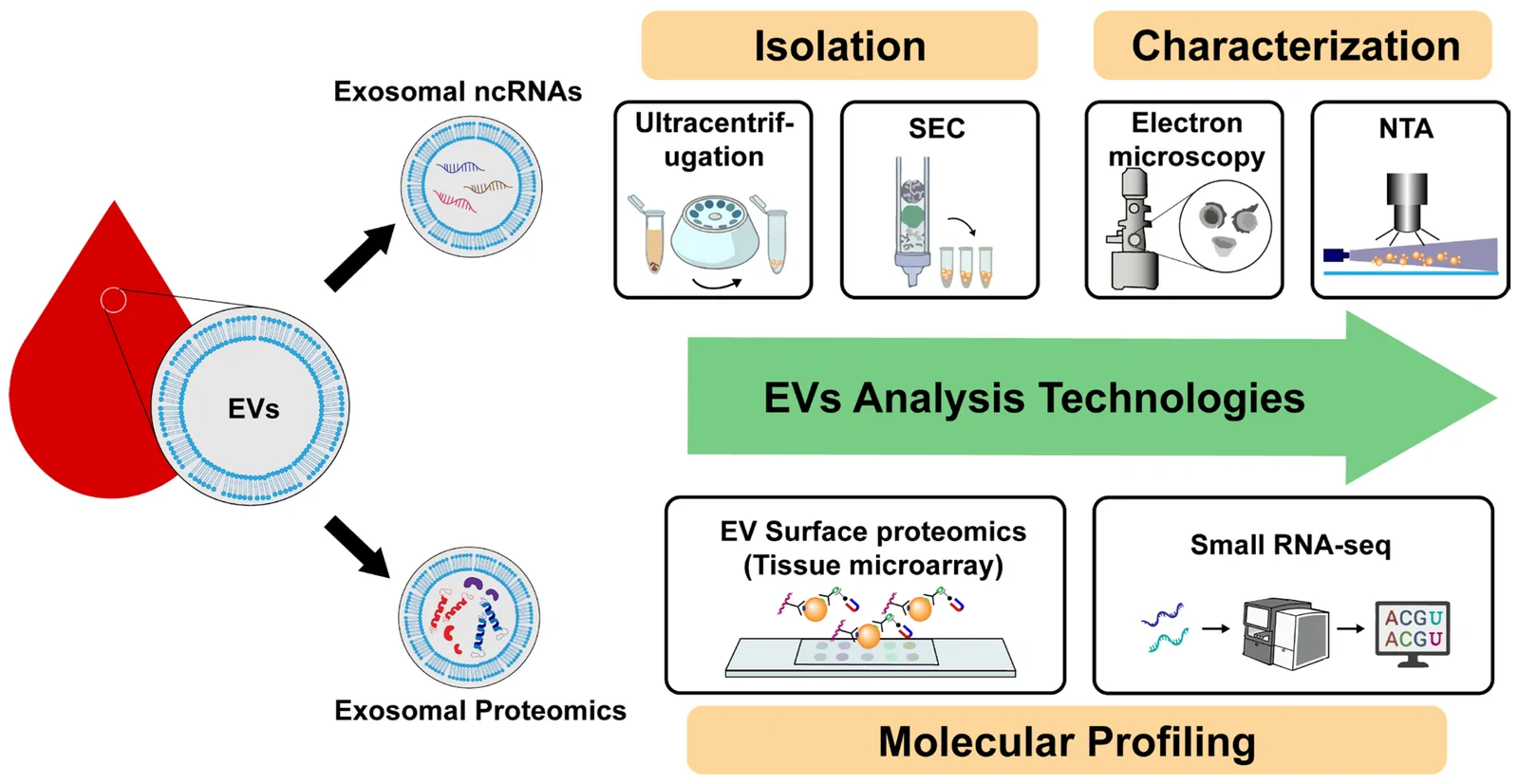
This schematic illustrates the main analytical features and analysis technologies of EVs. The analytical features include exosomal ncRNAs and exosomal proteomics. The analysis technologies are divided into isolation, characterization, and molecular profiling methods. Isolation methods include ultracentrifugation and size-exclusion chromatography (SEC). Characterization methods include electron microscopy and nanoparticle tracking analysis (NTA). Molecular profiling technologies include EV surface proteomics (tissue microarray) and small RNA sequencing (Small RNA-seq).

For MVI diagnostics, these platforms offer non-invasive discovery of vascular invasion biomarkers via ctRNA/EV-derived molecules, yet critical translation challenges persist: detecting ultra-low abundance MVI signatures, standardizing pre-analytical variables (especially EV isolation), validating specificity against background noise and non-MVI HCC, scaling complex protocols clinically, and conducting large prospective studies correlating liquid biopsy findings with histopathology. Specific detection methods can be found in Table 2. Classifications of current EV detection technologies are presented in Figure 5.

Recent advances in liquid biopsy have enabled increasing exploration of circulating biomarkers for the preoperative prediction of MVI in HCC. Existing studies indicate that ctDNA, CTCs, ctRNAs, and EVs reflect complementary aspects of tumor burden, vascular dissemination, and invasion-related molecular programs associated with MVI. However, the level of clinical evidence supporting individual biomarkers varies considerably across analyte classes and study designs. To address this heterogeneity, we propose an evidence-level classification framework that stratifies reported biomarkers according to the strength and directness of their MVI validation (Table 1): Level A, Level B, Level C, and Level D. This evidence-based framework reveals a notable translational gradient: ctDNA- and CTC-based markers have accumulated the highest level of evidence through direct histopathological correlation [57,58], whereas the majority of ctRNA and EV-derived biomarkers remain at exploratory evidence levels (C–D), indicating a pressing need for prospective validation studies with MVI-specific endpoints. The evidence-level classification of individual biomarkers is detailed in Table 1, and a systematic overview of available detection methodologies is provided in Table 2.

## 9. Pre-Analytical and Analytical Considerations

The reliability and comparability of liquid biopsy-based MVI prediction depend critically on standardization across the entire workflow, from blood collection to data analysis. Current studies exhibit substantial methodological heterogeneity that limits cross-study comparisons and impedes clinical translation.

Blood collection and processing. The choice of blood collection tube significantly affects cfDNA yield and stability. EDTA tubes require processing within 2–4 h to prevent leukocyte lysis and genomic DNA contamination, whereas specialized cell-free DNA blood collection tubes (BCTs) stabilize cfDNA for up to 48 h at room temperature through proprietary preservatives [122]. For CTC analysis, the time from venipuncture to processing affects cell viability and surface marker expression; processing delays exceeding 4 h have been associated with reduced CTC recovery and altered phenotypic marker profiles [123]. For EV studies, the collection tube type, anticoagulant, and processing delay influence vesicle integrity, concentration, and cargo composition [124]. Standardization of collection and initial processing protocols is therefore a prerequisite for cross-study reproducibility.

Centrifugation and storage. Standardized two-step centrifugation (typically 1600× *g* for 10 min at 4 °C followed by 16,000× *g* for 10 min at 4 °C) is recommended for plasma preparation to minimize residual cellular debris, though protocols vary substantially across published MVI studies [125]. Plasma storage at −80 °C is standard, but freeze–thaw cycles degrade ctRNA and may alter EV morphology and cargo. A maximum of one freeze–thaw cycle should be maintained, and aliquoting upon initial processing is strongly recommended to avoid repeated thawing.

Extraction and isolation methods. For cfDNA, silica column-based kits and magnetic bead-based methods show variable recovery efficiency depending on fragment size, with potential bias toward shorter or longer fragments [126]. EV isolation methods differ substantially: ultracentrifugation yields high-purity preparations but is time-intensive and operator-dependent; SEC provides relatively pure plasma EVs rapidly but at lower yield; polymer-based precipitation offers convenience but co-isolates non-EV contaminants including lipoproteins and protein aggregates; immunoaffinity capture achieves high specificity for selected surface markers but may miss subpopulations lacking the target antigen [127]. These methodological differences directly affect downstream molecular profiling and must be carefully considered when comparing results across studies.

Detection platforms and bioinformatics. Digital PCR offers high sensitivity for known targets with absolute quantification but limited multiplexing capacity. NGS enables broad genomic, transcriptomic, or methylomic profiling but introduces technical variability related to sequencing depth, library preparation protocols, alignment algorithms, and variant-calling pipelines [128]. The choice of platform and bioinformatics workflow can substantially influence the detection of low-abundance MVI-associated signals, and inter-laboratory harmonization efforts are urgently needed.

Hemolysis. Hemolysis during blood collection or processing releases erythrocyte-derived miRNAs and other cellular RNAs that can dominate sequencing libraries and mask tumor-derived signals. Spectrophotometric assessment of hemolysis (e.g., absorbance measurement at 414 nm for hemoglobin) should be routinely reported in ctRNA and EV studies, and samples with moderate-to-severe hemolysis should be excluded or analyzed with appropriate correction algorithms [129].

In summary, until these pre-analytical and analytical variables are harmonized through consensus protocols and inter-laboratory validation studies, the clinical implementation of liquid biopsy-based MVI prediction will remain aspirational. Future studies should adopt standardized reporting frameworks, such as the BIOSPECIMEN Reporting for Improved Study Quality (BRISQ) guidelines, to facilitate meaningful data integration and meta-analysis [130].

## 10. Liquid Biopsy Models for MVI Prediction

The use of liquid biopsy for the preoperative risk assessment of MVI has gradually garnered attention due to its potential value in clinical management. However, its primary value lies in biologically grounded risk stratification, rather than in directly guiding clinical management selection. Quantitative ctDNA metrics enable the classification of high- and low-risk MVI populations [57], while CTCs, ctRNAs, and EVs provide complementary evidence of vascular invasion and aggressive tumor behavior. It is important to recognize that the individual biomarkers feeding into these models differ substantially in their evidence strength for MVI prediction, as categorized in Table 1; models incorporating biomarkers with direct MVI validation should be weighted differently in clinical interpretation than those relying primarily on indirect or mechanistic associations.

Although these biomarkers demonstrate a significant association with MVI, the majority of current studies still rely on a single-marker approach. Such strategies are constrained by tumor heterogeneity, inter-individual variability, and the low abundance of circulating analytes, resulting in suboptimal predictive performance. To address these limitations, recent studies have increasingly adopted integrative strategies. These strategies involve combining multiple liquid biopsy markers or integrating clinicopathological and imaging data to develop more robust predictive models [131]. Such methods improve the accuracy and reliability of preoperative MVI assessment.

This section reviews comprehensive models based on liquid biopsy, which can be categorized into three types: multi-marker liquid biopsy models, liquid biopsy–clinicopathological integration models, and liquid biopsy–imaging integration models. By moving beyond single biomarkers to enhance the accuracy of MVI risk stratification, these models provide a quantitative reference for risk assessment and enable intuitive risk stratification: MVI-negative patients are defined as low-risk, while MVI-positive patients are categorized as high-risk per clinical guidelines. This stratification may provide a quantitative reference basis for personalized clinical management of HCC.

Before discussing specific models, it is useful to classify current liquid biopsy-based prediction models according to their stage of clinical readiness:

Exploratory models: Derived from single-center retrospective data with internal validation only (e.g., cross-validation or split-sample validation). While reported AUC values provide proof-of-concept, these models are not yet suitable for clinical application due to unproven generalizability.

Validation candidates: Models that have undergone external validation in one or more independent cohorts. These warrant prospective evaluations but have not yet demonstrated clinical utility in real-world settings.

Potential clinical tools: Models validated in multicenter prospective studies, with decision-curve analysis demonstrating clinical net benefit, predefined and validated risk thresholds, and evidence of transportability across populations and healthcare settings. At present, no liquid biopsy-based MVI prediction model has reached this level of maturity.

This framework provides the basis for critically evaluating the representative models discussed in the following subsections. Reported performance metrics should be interpreted in light of each model’s stage of development, while recognizing that the models highlighted here are intended to illustrate the current state of the field rather than provide an exhaustive catalog of all published studies.

### 10.1. Multi-Marker Liquid Biopsy Models

Current multi-marker liquid biopsy models for MVI prediction are predominantly built on conventional circulating biomarkers, including tumor-related proteins such as AFP and PIVKA-II, as well as inflammatory mediators and other blood-derived indicators. Consistent with this approach, proteomic analysis of 50 HCC patients identified three plasma biomarkers, TALDO1, PDIA3, and PGK1, that were significantly upregulated in MVI-positive cases (FDR-adjusted *p* < 0.05). A machine learning model (PRIM) built on these markers demonstrated strong discriminatory performance, with AUC values ranging from 0.78 to 0.99 across three independent cohorts [132]. Given that PRIM was developed and validated across four independent medical centers with geographically distinct cohorts (Harbin, Changchun, and Shenyang), this model may be considered a validation candidate. However, all cohorts were retrospectively recruited within Chinese populations, and prospective evaluation in ethnically and geographically diverse settings is warranted before clinical application. Similarly, serum CXCL8, CXCL9 and CXCL13 were significantly elevated in MVI-positive HCC patients and positively correlated with Child–Pugh scores and AFP levels. The combined chemokine panel achieved robust MVI prediction, with an AUC of 0.86, sensitivity of 74.39% and specificity of 85.71% [133]. This model should currently be regarded as an exploratory model, as validation was limited to an internal retrospective cohort without independent external confirmation.

Although multi-marker liquid biopsy models improve MVI prediction by integrating diverse circulating biomarkers, current models predominantly remain at the exploratory stage, relying on retrospective single-center cohorts with internal cross-validation [133]; one model (PRIM) has advanced to validation-candidate status through multicenter external validation [132]. The ratio of candidate features to MVI-positive cases is often high, raising the risk of overfitting, whereby models capture dataset-specific noise rather than generalizable biological signals. Few studies report calibration metrics (e.g., calibration plots and Brier scores) or perform decision-curve analysis to quantify clinical net benefit across a range of risk thresholds [134]. The optimal probability threshold for classifying patients as high-risk MVI versus low-risk MVI has not been standardized across studies, and the transportability of these models to different patient populations, including those with different HCC etiologies, geographic regions, and healthcare settings, remains untested. Furthermore, current models rely heavily on protein-based features, particularly high-throughput proteomics, which are costly, technically variable, and lack standardization, while the incremental predictive value of adding such complex biomarkers to simpler models based on routine clinical variables has rarely been formally quantified through net reclassification improvement (NRI) or integrated discrimination improvement (IDI) analyses [135]. Emerging biomarkers such as ctDNA, ctRNA, and EVs may provide complementary information and further enhance diagnostic accuracy when integrated into existing models.

### 10.2. Liquid Biopsy–Clinicopathological Integration Models

Integrated models combining liquid biopsy biomarkers with clinicopathological features leverage established clinical variables to improve MVI risk stratification and inform individualized therapeutic clinical management [57,136].

A representative model incorporating ctDNA allele frequency with tumor size achieved strong predictive performance, with an AUC of 0.92, sensitivity of 89.7% and specificity of 80.0% in the training cohort, and 78.6% sensitivity and 81.8% specificity in the validation cohort. In addition to diagnostic accuracy, this model also showed value in predicting recurrence-free survival [57]. This model should currently be regarded as an exploratory model, as the training and validation cohorts were derived from a 2:1 random split of a single-center cohort (*n* = 73) without independent external validation. Similarly, nomogram-based models integrating clinicopathological factors, such as tumor diameter and HBV status, with serum biomarkers including AFP, AFP-L3%, PIVKA-II, and CA125 demonstrated stable performance, with AUCs of 0.806 and 0.818 in development and validation cohorts, respectively [137]. This model should currently be regarded as an exploratory model, as the development and validation cohorts were derived from a 3:1 random split of a single-center retrospective dataset (*n* = 1027), without independent external validation. Other models combining tumor differentiation, tumor size ≥ 5 cm, and blood-based markers such as PIVKA-II ≥ 90 mAU/mL and platelet-to-lymphocyte ratio ≥150 achieved AUCs of 0.81 and 0.83 in training and validation sets, with good calibration and clinical net benefit [136]. This model should currently be regarded as an exploratory model, as the training and validation sets were derived from a 7:3 random split of a single-center retrospective cohort (*n* = 300) without independent external validation.

Overall, these models combine the robustness of clinicopathological variables with the non-invasive advantages of liquid biopsy, improving preoperative MVI assessment. However, all currently published models in this category remain at the exploratory stage, having been developed and internally validated within single-center retrospective cohorts [57,136,137]. While the integration of clinicopathological variables generally enhances model robustness compared with biomarker-only approaches, several important limitations persist. Most models are derived from surgical series with inherent selection bias, as patients deemed resectable represent a subset with more favorable tumor biology and preserved liver function, limiting generalizability to the broader HCC population [138]. External validation in geographically and etiologically distinct populations remains limited; a model developed in an HBV-endemic Asian cohort may not perform equivalently in a Western cohort with predominantly metabolic-associated HCC [139]. Moreover, the incremental value of liquid biopsy features over established clinical predictors, including tumor size and AFP, has not been systematically quantified [140]. The stability of model features across studies is also low, with different combinations of biomarkers and clinicopathological variables emerging from different cohorts, suggesting that feature selection may be driven more by dataset-specific associations than by consistent biological signals. In addition, current models are often constrained by retrospective single-center designs, high costs, lack of standardization, and variability in selected model features. While ctDNA-based models have demonstrated clinical value, emerging biomarkers such as ctRNA and EVs may provide complementary biological information and further enhance predictive performance. Their integration with clinicopathological features may facilitate the development of more accurate, reproducible, and standardized models for preoperative MVI prediction.

### 10.3. Liquid Biopsy–Imaging Integration Models

Liquid biopsy integrated with imaging features combines molecular and morphological information, enabling accurate preoperative prediction of MVI in HCC and supporting imaging-guided clinical management [141,142].

A representative scoring model incorporating CTCs ≥3/3.2 mL, AFP ≥158 ng/mL, and des-γ-carboxy prothrombin (DCP) ≥ 178 mAU/mL with imaging features such as tumor diameter ≥ 59 mm and unsmooth margins achieved strong performance, with an AUC of 0.922, sensitivity of 86.49%, and specificity of 90.20% [141]. This model should currently be regarded as an exploratory model, as the analysis was conducted on a single-center retrospective cohort (*n* = 227) without a dedicated validation cohort or external validation. Similarly, a nomogram combining AFP >800 ng/mL with MRI features, including arterial phase edge enhancement, incomplete capsule, and intratumoral vascular enhancement, showed stable efficacy, with AUCs of 0.871 and 0.878 in training and validation cohorts [143]. This model should currently be regarded as an exploratory model, as validation was limited to a 1:1 random split of a single-center retrospective cohort (*n* = 236), supplemented by 5-fold internal cross-validation, without independent external validation. Another radiogenomic nomogram integrating plasma miR-21 with MRI-derived tumor size and radiomic score also demonstrated high accuracy (AUC = 0.900, sensitivity = 0.970, specificity = 0.722) [142]. This model should currently be regarded as an exploratory model, as the training (*n* = 116) and validation (*n* = 52) cohorts were derived from a single-institution retrospective dataset without independent external validation. Other predictive models based on gadobenate-enhanced MRI combined AFP with features such as peritumoral hypointensity and atypical enhancement patterns, yielding AUCs around 0.83 [144]. This model should currently be regarded as an exploratory model, as validation was performed using 4-fold internal cross-validation on a single-center retrospective cohort (*n* = 164) without independent external validation.

These models fully leverage the complementary strengths of liquid biopsy and imaging, enabling the non-invasive and reproducible assessment of MVI. However, all currently published imaging–liquid biopsy integration models remain at the exploratory stage, having been derived from single-center retrospective cohorts with internal validation only [141,142,143,144]. Direct comparative data indicate that integrated models combining CTCs with imaging perform best in predicting MVI (AUC = 0.922), followed by ctDNA-clinicopathological models, while single-biomarker assays show the lowest predictive accuracy [57,133,141]; however, these comparisons are limited by the exploratory nature of the underlying studies, and relative rankings may not be reproducible in external validation settings. Notably, multi-omics integration represents a promising strategy for addressing the limitations of single-biomarker approaches, although its added value over simpler models must be demonstrated through prospective studies with predefined analysis plans. By combining imaging and liquid biopsy data, these models have the potential to improve preoperative MVI prediction and enable more individualized risk assessment. However, imaging–liquid biopsy integrated models also introduce additional methodological and practical complexity. Radiomics feature extraction remains non-standardized across CT and MRI platforms, reconstruction algorithms, and segmentation protocols, limiting reproducibility across institutions [145]. The relative contribution of imaging versus liquid biopsy features to overall model performance is rarely reported through formal feature-importance or ablation analyses, making it difficult to determine whether the increased cost and complexity of multimodal data collection are justified. Furthermore, many imaging features incorporated into these models, such as irregular tumor margins and peritumoral enhancement, are themselves established correlates of MVI, raising the question of whether liquid biopsy biomarkers provide independent predictive information beyond high-quality imaging [146]. Although current models integrating CTCs, miRNAs, and serum markers have demonstrated clinical utility, emerging biomarkers such as ctDNA and EVs may offer complementary molecular information with higher biological resolution. Their incorporation into imaging-based frameworks may further refine multimodal characterization and improve predictive performance. Nevertheless, prospective studies with pre-specified imaging acquisition protocols, standardized liquid biopsy collection time points, predefined analytical workflows, and blinded MVI endpoint adjudication are required to determine the true incremental value and clinical applicability of these integrated models [147].

## 11. Therapeutic Strategies for MVI-Positive HCC Patients

It is important to note that liquid biopsy-based prediction of MVI has not yet been validated for treatment selection in routine clinical practice. Most studies discussed in this section stratified outcomes according to histopathologically confirmed postoperative MVI status rather than preoperative liquid biopsy-derived risk estimates. Consequently, the clinical implications outlined below should be viewed as a potential framework for future MVI-guided management, pending prospective validation, establishment of clinically actionable risk thresholds, and incorporation into consensus guidelines. Until such evidence becomes available, treatment decisions should continue to rely on established clinical, radiological and pathological criteria.

According to the Chinese Guidelines for the Diagnosis and Treatment of Primary Liver Cancer 2022 (CLCG 2022) [148], MVI is a key determinant of the biological heterogeneity and aggressiveness of HCC and is strongly associated with postoperative recurrence risk and long-term prognosis. Accordingly, routine assessment of MVI is recommended as part of postoperative pathological evaluation.

The principal translational value of liquid biopsy approaches lies in their potential to provide preoperative estimation of MVI risk. If validated in large prospective cohorts, these biomarkers could shift MVI assessment from the postoperative to the preoperative setting, enabling earlier risk stratification and providing complementary information for multidisciplinary treatment planning.

This section therefore summarizes current evidence regarding the incorporation of MVI status, or future MVI risk estimates, into surgical and perioperative management strategies for HCC. Particular emphasis is placed on surgical approach, resection margin strategy, liver transplantation, and perioperative locoregional therapies, with the goal of advancing risk-adapted and MVI-informed clinical decision-making.

### 11.1. Hepatectomy

Hepatectomy, the surgical removal of the liver portion containing the tumor, is the most widely used potentially curative treatment for early HCC patients with preserved liver function and no significant portal hypertension [149]. The type and extent of resection, including anatomical resection (AR), non-anatomical resection (NAR), and surgical margin width, exert a crucial influence on postoperative outcomes. This influence is mediated through their effects on the clearance of micrometastases and the preservation of the hepatic parenchyma. MVI is a significant predictor of recurrence and poor prognosis [150,151], and it may influence the formulation of surgical strategies. For MVI-positive tumors, wider margins combined with AR may be beneficial, whereas for MVI-negative tumors, parenchyma-sparing NAR can achieve comparable outcomes. Preoperative MVI assessment is crucial for formulating surgical strategies, balancing tumor control with liver function preservation, and optimizing survival rates.

#### 11.1.1. Surgical Margin Width Selection

In HCC, surgical margins are generally classified as narrow (≤0.5 cm) and wide (>1.0 cm). The 2022 CLCG guidelines recommend a personalized approach to surgical margins in cases of HCC with MVI, given its strong association with postoperative recurrence [148]. This approach is biologically motivated by evidence that MVI can extend beyond the macroscopic tumor boundary [152], raising concerns regarding residual microscopic lesions following resection.

Multiple studies have demonstrated that a wider margin (generally >0.5–1.0 cm) may remove peritumoral microscopic metastases, thereby reducing recurrence and improving survival [153,154,155]. However, recent data suggest that when negative margins and high AR rates are achieved, long-term survival rates may not differ significantly across margin width categories (≤0.3 cm, 0.3–1.0 cm, >1.0 cm), regardless of MVI status [156]. This suggests that, at least in certain cases, outcomes in MVI-positive tumors, particularly larger ones, may be driven more strongly by the systemic or intrahepatic micrometastatic burden than solely by local surgical margin control.

Clinically, the practice of employing a wide margin is frequently constrained by background liver disease. In China, more than 80% of HCC patients have cirrhosis [157], and preservation of functional liver reserve often limits the feasibility of performing wide margins. In summary, although the survival benefit of wider resection margins remains controversial, current evidence suggests that MVI status may serve as an important modifying factor in surgical margin planning, helping to balance oncological benefit against preservation of hepatic functional reserve; however, integration of preoperative MVI risk prediction into routine surgical decision-making awaits further prospective validation.

#### 11.1.2. Anatomical Resection (AR) and Non-Anatomical Resection (NAR)

AR and NAR are two fundamental surgical approaches for HCC. AR involves the resection of the tumor and the corresponding portal vein territory to eliminate potential portal vein spread, whereas NAR focuses on achieving tumor-free margins while preserving functional parenchyma, without regard for segmental anatomy [158,159]. According to CLCG 2022, The choice between AR and NAR should be an individualized decision based on hepatic functional reserve and tumor biology [148]. In this context, MVI status or the preoperative risk of MVI, serves as a critical factor in adjusting surgical strategy, favoring AR when liver function is adequate, while supporting the use of NAR in patients with limited liver reserve or those for whom liver parenchyma preservation is a priority.

Given that MVI reflects early vascular dissemination, AR is theoretically expected to confer a survival advantage in tumors. However, clinical evidence remains conflicting. AR offers limited survival benefit in patients without MVI or with well-differentiated tumors, suggesting its value may be greater in high-risk MVI patients [160]. Other studies further indicate that AR combined with a wide margin (>1 cm) can synergistically improve outcomes in high-risk MVI patients [161,162]. Nevertheless, this benefit has not been consistently validated in the broader MVI cohort [163,164], and the effect may be constrained by factors such as tumor size or impaired liver function [154,165]. By preserving parenchyma, NAR is often more suitable for cirrhotic patients, particularly those with limited hepatic reserve [166]. However, in patients with MVI, NAR constitutes an independent risk factor for recurrence, as it may fail to eradicate micrometastases disseminating via tumor-associated portal vein branches. This risk is particularly pronounced when NAR is performed with narrow resection margins, as this approach is associated with the highest rate of early recurrence among all surgical methods [162]. Nonetheless, for MVI-positive patients with significant hepatic dysfunction, NAR may still be the only viable surgical option.

In summary, these findings indicate that MVI should be incorporated into clinical management as a stratification factor, rather than serving as an absolute determinant for selecting AR over NAR. Its pivotal role lies in refining patient stratification and supporting individualized surgical planning, particularly when balancing oncologic benefits against hepatic functional reserve. Notably, these considerations are based on histologically confirmed MVI following resection. The extent to which liquid biopsy-based preoperative MVI risk prediction can inform the AR-versus-NAR decision awaits dedicated prospective evaluation.

### 11.2. Liver Transplantation (LT)

Liver transplantation (LT) is considered the most definitive treatment for selected HCC patients, as it removes both tumors and diseased liver parenchyma [167]. Based on treatment sequence, LT can be classified into primary LT and salvage LT (SLT). Primary LT is performed as the first-line curative therapy, whereas SLT is reserved for tumor recurrence or liver failure following prior liver resection. In the context of limited donor organ availability, appropriate transplant candidate selection is essential to optimize post-transplant outcomes. MVI, a strong predictor of early recurrence and poor survival, has a significant impact on prognosis after both primary LT and SLT. Currently, transplant eligibility is largely determined by morphology-based criteria, including the Milan, UCSF, and Kyoto 5-5-500 frameworks [168,169,170]. Incorporating MVI into these criteria may further refine risk stratification and inform optimal transplant strategy.

#### 11.2.1. Impact of MVI on Primary Liver Transplantation Outcomes

MVI independently predicts poor outcomes after LT, being associated with higher recurrence and reduced survival [171,172,173,174]. However, risk stratification studies have shown that the impact of MVI varies significantly among different patient subgroups. An East–West collaborative study reported that although living donor liver transplantation (LDLT) and liver resection (LR) can achieve comparable 5-year survival in early-stage HCC, MVI appears to exert a more detrimental impact on LDLT outcomes [175]. Other evidence suggests that selected low-risk MVI patients may still achieve favorable results after LT or AR [176]. In very early tumors (<2 cm) [177] or in patients meeting the up-to-7 criteria [178], post-LT outcomes can remain acceptable despite MVI, underscoring the heterogeneity of its clinical effect.

Thus, MVI should not be regarded as an absolute contraindication to LT. Instead, it should be integrated into a multifactorial evaluation incorporating tumor burden, imaging characteristics, biological behavior, and liver function. More refined stratification models are still needed to identify which MVI-positive patients can derive meaningful benefit from LT while maintaining acceptable recurrence risk. Whether liquid biopsy-derived MVI risk stratification, as opposed to histologically confirmed MVI, can contribute meaningfully to LT candidate selection has not been studied and represents an important area for future investigation.

#### 11.2.2. Impact of MVI on Salvage Liver Transplantation (SLT)

SLT offers a strategy to conserve donor livers by performing LR first and reserving transplantation for recurrence. Initial LR is feasible without compromising subsequent LT safety and can provide long-term survival if recurrence remains within transplant criteria [179]. However, MVI severely limits SLT efficacy [175]. Patients with MVI or satellite nodules frequently experience early and aggressive recurrence, progressing beyond transplant eligibility before SLT can be performed [179,180].To mitigate this limitation, the ab initio LT strategy proposes listing patients with high-risk pathology (MVI/satellites) immediately post-LR. Given early recurrence (<6 months) indicates aggressive biology, listing after ≥6-month observation is recommended. Preliminary data suggests this approach may improve survival in selected patients [181].

In summary, SLT remains feasible for selected patients, but its utility is constrained by the rapid progression associated with MVI. Early incorporation of MVI status into post-resection surveillance may help identify patients who require closer monitoring, earlier transplant listing, or alternative strategies. Future studies should evaluate whether preoperative liquid biopsy-based MVI prediction can refine SLT candidate selection beyond histopathological criteria.

### 11.3. Transarterial Chemoembolization (TACE)

TACE is a widely used locoregional therapy for intermediate-stage HCC and remains the standard of care for patients who are unsuitable for curative treatments [182]. In contrast, its role as a neoadjuvant approach in resectable HCC is limited, and current guidelines do not recommend routine preoperative TACE [183]. Although response to neoadjuvant TACE may provide insight into tumor biology and has been associated with prognosis, this response alone is insufficient for risk stratification. MVI consistently remains an independent adverse prognostic factor, underscoring the need for more refined, risk-adapted clinical management strategies [184]. In this context, attention has shifted toward the potential role of postoperative adjuvant TACE (PA-TACE) in improving outcomes after hepatectomy. Although PA-TACE has shown variable benefit overall [185], substantial evidence demonstrates clear efficacy in high-risk subgroups characterized by factors such as large tumor size, multinodularity, macrovascular invasion, poor differentiation, or MVI [186,187]. Stratified analyses further reveal that PA-TACE improves survival and reduces early recurrence specifically in patients with high-risk MVI profiles, while offering limited benefit in low-risk populations [188]. Therefore, MVI serves as a key determinant for selecting patients who are most likely to benefit from PA-TACE after hepatectomy.

The presence of histologically confirmed MVI has been associated with differential benefit from adjuvant TACE in retrospective analyses, suggesting a potential role for MVI risk stratification in guiding perioperative TACE decisions. While response to neoadjuvant TACE may reflect tumor biology, evidence indicates that PA-TACE has been associated with survival and recurrence benefits in patients with high-risk MVI profiles in retrospective studies, supporting the concept of risk-adapted adjuvant therapy. Prospective studies are needed to validate these findings and to determine whether preoperative liquid biopsy-based MVI prediction can identify patients most likely to benefit from adjuvant TACE.

Overall, current evidence highlights the important role of MVI in the clinical management of HCC. Histologically confirmed MVI is increasingly recognized as a marker of aggressive tumor biology and a higher risk of recurrence, and may contribute to risk stratification across different treatment settings. In surgical management, MVI status can be considered alongside other clinical and tumor-related factors when determining resection strategies and balancing oncological radicality against liver function preservation. In the context of transplantation, MVI may provide additional prognostic information that could help refine patient selection and post-resection listing strategies. Furthermore, MVI has been associated with differential responses to perioperative locoregional therapies and may help identify patients who are more likely to benefit from postoperative adjuvant TACE. However, these considerations are largely derived from studies using histologically confirmed MVI, and their applicability to preoperative MVI prediction models requires further prospective validation. Overall, incorporating MVI into a multidisciplinary and risk-adapted management framework may contribute to more individualized treatment planning and improved long-term outcomes in selected patients with HCC.

## 12. Translational Pathway Toward Clinical Implementation

The translation of liquid biopsy-based MVI prediction from research to clinical practice requires a structured, staged pathway. Stage 1 (Retrospective validation): Candidate biomarkers and models identified in discovery cohorts must undergo external validation in independent retrospective cohorts with histologically confirmed MVI to establish generalizability. Stage 2 (Prospective observational studies): Pre-specified biomarker panels, standardized collection and analysis protocols, and blinded MVI endpoint adjudication should be employed in prospective cohorts to evaluate real-world predictive performance and to establish optimal risk thresholds. Stage 3 (Clinical utility assessment): Studies must evaluate whether liquid biopsy-informed MVI risk stratification actually changes treatment decisions and, more importantly, improves patient outcomes. Ideally, randomized designs comparing liquid biopsy-informed management versus standard-of-care management should be conducted. Stage 4 (Threshold standardization): Predefined, validated risk thresholds must be established for each clinical decision context (surgical margin width, transplant eligibility, adjuvant therapy selection), as a single MVI-risk cutoff is unlikely to serve all clinical scenarios. Stage 5 (Guideline integration): Multidisciplinary consensus guidelines incorporating liquid biopsy-based MVI risk stratification should be developed through international collaboration before routine clinical implementation. This translational pathway, while demanding, provides a roadmap for the responsible and evidence-based integration of liquid biopsy into the preoperative management of HCC.

## 13. Discussion

MVI remains a pivotal determinant of early recurrence and long-term survival in HCC, yet its assessment continues to rely largely on postoperative histopathology and indirect imaging surrogates. As summarized in this narrative review, liquid biopsy offers a minimally invasive approach for preoperative MVI risk stratification by capturing tumor-derived molecular and cellular signals associated with vascular invasion and early dissemination. Current evidence indicates that ctDNA, CTCs, ctRNA and EVs provide complementary insights into distinct aspects of MVI-associated tumor biology, collectively supporting their potential role in improving preoperative risk assessment.

Accumulating evidence indicates that various liquid biopsy analyses reflect complementary aspects of MVI-associated tumor biology. ctDNA profiling, including somatic mutations, methylation profiles, and fragmentation features, enables the capture of genomic and epigenetic alterations associated with aggressive tumor behavior and early recurrence. CTCs, particularly mesenchymal and hybrid EMT phenotypes, provide direct cellular evidence of vascular intravasation and invasive potential, while quantitative CTC burden serves as a strong predictor of MVI. In parallel, ctRNA and EV–derived biomarkers expand the molecular landscape by reflecting invasion-associated regulatory programs and intercellular communication. Overall, these findings suggest that MVI may reflect a broader continuum of tumor invasion and dissemination, which can be partially captured by circulating biomarkers. However, current evidence primarily demonstrates associations rather than direct causality or relationships specific to MVI, and therefore should be interpreted cautiously. To facilitate an evidence-aware reading of the literature, we have systematically classified all reviewed biomarkers into four evidence tiers in Table 1, ranging from direct MVI histopathological validation (Level A) to mechanistic plausibility without clinical MVI data (Level D). This classification reveals that Level A evidence is concentrated in a relatively small subset of studies, primarily those evaluating ctDNA VAF, mSEPT9, CTC enumeration, M-CTC counts, serum miR-125b, circSETD3, S100P, and exosomal PRPSAP1. The majority of remaining biomarkers provide indirect (Level B–C) or purely mechanistic (Level D) evidence. This evidence imbalance represents a critical knowledge gap that should guide future prospective study design.

A key contribution of this review lies in its MVI-centric, integrative, and translation-oriented framework, which incorporates an explicit evidence-strength stratification to distinguish biomarkers with direct histopathological validation for MVI from those supported primarily by indirect clinical associations or mechanistic evidence. Unlike previous reviews that have primarily discussed liquid biopsy in HCC from diagnostic or prognostic viewpoints, this review examines ctDNA, CTCs, ctRNA, and EVs in relation to MVI as a unifying biological and clinical endpoint. Beyond integrating evidence across diverse biomarker categories and detection platforms, we further connect liquid biopsy findings with multi-omics predictive models. By combining complementary molecular signals within an evidence-graded framework, we aim to address the inherent limitations of single-biomarker approaches and provide a more rigorous assessment of MVI prediction. This integrative strategy highlights the potential of liquid biopsy-based multi-omics profiling for preoperative MVI evaluation, moving beyond isolated biomarker associations toward clinically actionable risk stratification.

Importantly, this review also highlights several limitations of the current evidence base. Most studies evaluating liquid biopsy biomarkers for MVI are retrospective, single-center investigations with substantial methodological heterogeneity in sample processing, analytical platforms and reporting criteria. Emerging multi-omics models have shown encouraging performance; however, their clinical translation is constrained by limited cohort sizes, a lack of external multicenter validation, undefined clinical cut-offs and the absence of standardized pre-analytical and analytical workflows. Furthermore, a notable knowledge gap is the absence of head-to-head comparative studies evaluating different liquid biopsy modalities—ctDNA, CTCs, ctRNA, and EVs—for MVI prediction within the same patient cohort. Without such comparative data, the relative clinical value and complementarity of each analyte class remain uncertain, and rational selection or prioritization of biomarkers for multi-analyte panels is precluded. Future studies should ideally collect and analyze multiple liquid biopsy fractions from the same blood draw in prospective cohorts with histologically confirmed MVI as the primary endpoint, enabling direct performance comparisons and assessment of additive predictive value.

Several limitations of this review should also be acknowledged. In addition, this review deliberately focused on four major liquid biopsy analyte classes that currently provide the most substantial evidence base for MVI assessment. Consequently, other emerging liquid biopsy approaches, including EV–derived metabolomic, proteomic, and other multi-omics signatures, were not systematically reviewed. Although these fields remain relatively underexplored in the context of MVI, they may provide additional biological insights and represent important directions for future investigation. As a narrative synthesis, it does not follow a formal systematic-review methodology and may therefore be subject to selection bias despite efforts to comprehensively evaluate the available literature. No quantitative meta-analysis was performed, precluding formal assessment of pooled diagnostic performance and between-study heterogeneity. In addition, publication bias may favor studies reporting positive associations, potentially inflating estimates of biomarker performance, while the rapid evolution of liquid biopsy technologies means that some recently developed high-sensitivity platforms and multi-analyte approaches may not be fully represented.

From a clinical perspective, liquid biopsy and its integration into multi-omics predictive models should not be regarded as a replacement for histopathology, but rather as a tool for biologically informed preoperative risk stratification. Identification of patients at high risk of MVI via validated multi-omics models may, following prospective clinical validation, support more precise risk-adapted management strategies. Importantly, the clinical utility and reliability of such models are likely to depend on the evidence strength of their constituent biomarkers, with models incorporating biomarkers directly validated against histopathological MVI expected to provide more robust clinical decision support than those based primarily on indirect or mechanistic associations. However, as outlined in the Translational Pathway, several stages of validation, including external cohort replication, prospective observational studies, clinical utility trials, and threshold standardization, must be completed before liquid biopsy-based MVI prediction can inform treatment selection, surgical planning, or transplant eligibility.

Looking ahead, progress in this field will necessitate large-scale, multicenter prospective studies, following the staged Translational Pathway, to validate liquid biopsy-based MVI signatures and multi-omics models, standardize end-to-end analytical pipelines through inter-laboratory harmonization, and define clinically actionable, context-specific risk thresholds. Integration of multi-analyte liquid biopsy data with standardized imaging features and clinicopathological variables will further enhance predictive accuracy, but must be accompanied by rigorous demonstration of incremental clinical value. By consolidating current evidence, bridging biomarkers with multi-omics models, and outlining a structured translational pathway, this review provides a framework for advancing the preoperative assessment of MVI in HCC and promoting individualized, risk-adapted clinical management.

## Figures and Tables

**Table 2:** Blood Biomarker Analysis Technology.

Biomarker Type	Testing Type	Method	Year	Downstream Analysis Techniques	Assessment Metrics	Ref.
CtDNA	Targeted	ddPCR	2021	N/A	TERT promoter mutation with OS (HR = 0.74 (95% CI 0.34–1.61), *p* = 0.45)	[62]
CtDNA	Targeted	BEAMing	2008	N/A	Detectable vs. undetectable postoperative ctDNA, RFS (*p* = 0.006)	[63]
CtDNA	Targeted	Safe-SeqS	2016	N/A	Postoperative ctDNA with RFS (HR = 11 (95% CI 1.8–68), *p* = 0.001)	[64]
CtDNA	Targeted	TAm-Seq	2012	N/A	Detection of low-frequency mutations, sensitivity and specificity > 97%.	[65]
CtDNA	Untargeted	WES	2021	N/A	Co-occurrence of HRNR and TTN mutations with OS (HR = 1.60, *p* = 0.0196).	[68]
CtDNA	Untargeted	WGS	2023	N/A	Detectable ctDNA vs. undetectable; OS (HR = 7.69 (95% CI 2.09–28.27); *p* < 0.01)	[69]
CtDNA	Untargeted	WGBS	2022	N/A	CtDNA methylation panel, AUC = 0.967 (early); AUC = 0.971 (advanced)	[73]
CTC	Immunoaffinity	CellSearch system	2019	N/A	CTC ≥ 5/7.5 mL with median OS (HR = 2.7, *p* < 0.0001)	[74]
CTC	Immunoaffinity	IMNs	2023	N/A	Capture efficiency 88.3% (vs 70.5% control); nonspecific adsorption ↓5.5×, nonspecific cell binding ↓5.4×	[75]
CTC	Physical	Microcavity array	2021	N/A	positive CTCs (CTCs ≥ 10) predicts shorter OS (*p* = 0.025)	[76]
CTC	Physical	Dual-filtration system	2022	ScRNA-seq	Presence of Group 1 CT cells/clusters with shorter OS (39 vs. 384 days, *p* = 0.048).	[77]
CTC	Hybrid	Microfluidic Synergetic-Chip	2020	N/A	CTC detection and phenotype classification, Sensitivity = 97.8%, Specificity = 100%	[78]
CtRNA	Amplification	RT-ddPCR	2020	N/A	HCC EV Z-score (Early-stage HCC vs. Cirrhosis): AUC = 0.93 (95% CI 0.86–1.00); Sensitivity = 94.4%; Specificity = 88.5%	[79]
CtRNA	Amplification	d-SCOUT	2025	MRT-dPCR	molecular signatures derived from HCC CTCs (AUC = 0.950, sensitivity = 90.6%, specificity = 87.5%).	[80]
CtRNA	Amplification	qRT-PCR	2021	N/A	Circ_0011385 expression, Tumor size (*p* = 0.001); TNM stage (*p* = 0.007); OS (*p* < 0.05, high vs. low expression)	[81]
CtRNA	Capture-Seq	Target-RNAseq	2025	N/A	36-variant ctRNA panel identified all HCC patients (100% sensitivity vs. LC)	[82]
EVs	Isolation	Ultracentrifugation	2021	ELISA	EV-pIgR↑ in late-HCC (*p* < 0.001); Tumor-free survival shorter in EV-treated mice (log-rank *p* < 0.01)	[83]
EVs	Isolation	SEC	2021	LC–MS+RNA-seq	Proteomics reproducibility R^2^ = 0.98; small RNA reproducibility R^2^ = 0.96	[84]
EVs	Isolation	GlyExo-Capture	2024	Small RNA-seq	AFP + 5-miRNA model (HCC vs. non-HCC): AUC = 0.96	[85]
EVs	Isolation	Tidal microfluidic chip	2025	qRT-PCR	Bi-mRNAs (early-stage HCC vs. high-risk individuals): AUC = 0.846; bi-mRNAs (AFP-negative HCC vs. high-risk individuals): AUC = 0.926	[86]
EVs	Characterization	Electron microscopy	2022	qRT-PCR	MiR-483-5p (HCC vs. HC): AUC = 0.868; AFP + miR-483-5p (HCC vs. HC): AUC = 0.949	[87]
EVs	Characterization	NTA	2023	N/A	GPC3 (HCC vs. non-HCC): AUC = 0.79; AFP (HCC vs. non-HCC): AUC = 0.71; GPC3 + AFP (HCC vs. non-HCC): AUC = 0.72.	[88]
EVs	Characterization	Flow cytometry	2022	N/A	GPC3^+^ exosomes (HCC vs. non-cirrhotic vs. HC): 12.4% vs. 3.7% vs. 1.3%, *p* < 0.001.	[89]
EVs	Molecular profiling	EV Surface Proteomics (Tissue microarray)	2023	N/A	HCC EV ECG score (early-stage HCC vs. cirrhosis): AUROC = 0.95 (95% CI 0.90–0.99);	[90]
EVs	Molecular profiling	Small RNA-seq	2020	N/A	305 differentially expressed miRNAs (119↑, 186↓); *p* < 0.01, FC > 2	[91]

Abbreviations: ddPCR, droplet digital PCR; Safe-SeqS, safe-sequencing system; TAm-Seq, tagged amplicon deep sequencing; WES, whole exome sequencing; WGS, whole-genome sequencing; WGBS, whole genome methylomes sequencing; ScRNA-seq, single-cell RNA sequencing; IMNs, Immuno-magnetic nanoparticles system; RT-ddPCR, reverse-transcription droplet digital PCR; d-SCOUT, digital simultaneous cross-dimensional output and unified tracking; MRT-dPCR, multi-real-time digital PCR; qRT-PCR, quantitative real-time PCR; Target-RNAseq, targeted RNA sequencing; SEC, size-exclusion chromatography; LC-MS, liquid chromatography–mass spectrometry; Small RNA-seq, small RNA sequencing; NTA, nanoparticle tracking analysis.

## Data Availability

No new data were created or analyzed in this study. Data sharing is not applicable to this article.

## List of Abbreviations

AFP, alpha-fetoprotein; AJCC, American Joint Committee on Cancer; AR, anatomical resection; ARD, at-risk liver disease; BCLC, Barcelona Clinic Liver Cancer; BEAMing, beads, emulsion, amplification, and magnetics; CAP1, cyclase-associated protein 1; CAPP-Seq, cancer personalized profiling by deep sequencing; cfDNA, cell-free DNA; cfRNA, cell-free RNA; CHB, chronic hepatitis B; circRNAs, circular RNAs; CLCG 2022, Chinese Guidelines for the Diagnosis and Treatment of Primary Liver Cancer 2022; CT, computed tomography; ctDNA, circulating tumor DNA; ctRNA, circulating tumor RNA; CTCs, circulating tumor cells; DCP, des-γ-carboxy prothrombin; ddPCR, droplet digital PCR; d-SCOUT, digital simultaneous cross-dimensional output and unified tracking; E-CTCs, epithelial-phenotype circulating tumor cells; ELISA, enzyme-linked immunosorbent assay; EMT, epithelial–mesenchymal transition; EMT-CTCs, epithelial–mesenchymal transition-phenotype circulating tumor cells; EVs, extracellular vesicles; FSS, fluid shear stress; GPC-3, glypican-3; GVI, gross vascular invasion; HCC, hepatocellular carcinoma; ICI, immune checkpoint inhibitor; IGF2, insulin-like growth factor 2; IMNs, immunomagnetic nanoparticles system; ISET, isolation by size of epithelial tumor cells; LC, liver cirrhosis; LC-MS, liquid chromatography–mass spectrometry; LDLT, living donor liver transplantation; lncRNAs, long non-coding RNAs; LR, liver resection; LT, liver transplantation; MAF, mutant allele frequency; M-CTCs, mesenchymal-phenotype circulating tumor cells; MHB, methylation haplotype block; miRNAs, microRNAs; MRI, magnetic resonance imaging; MRT-dPCR, multi-real-time digital PCR; mSEPT9, methylated SEPT9; MVI, microvascular invasion; ncRNAs, non-coding RNAs; NAR, non-anatomical resection; NGS, next-generation sequencing; NMLD, non-malignant liver disease; NTA, nanoparticle tracking analysis; NuSS, number and spatial distribution of sampling sites; OS, overall survival; PA-TACE, postoperative adjuvant transarterial chemoembolization; PFS, progression-free survival; PIVKA-II, protein induced by vitamin K absence or antagonist-II; PVTT, portal vein tumor thrombus; qRT-PCR, quantitative reverse transcription PCR; RFS, recurrence-free survival; RT-ddPCR, reverse-transcription droplet digital PCR; Safe-SeqS, safe-sequencing system; ScRNA-seq, single-cell RNA sequencing; SEC, size-exclusion chromatography; SLT, salvage liver transplantation; Small RNA-seq, small RNA sequencing; sWGS, shallow whole-genome sequencing; TACE, transarterial chemoembolization; TAm-Seq, tagged amplicon deep sequencing; TAS, targeted amplification sequencing; Target-RNAseq, targeted RNA sequencing; TCS, targeted capture sequencing; TKI, tyrosine kinase inhibitor; VAF, variant allele frequency; VEGFA, vascular endothelial growth factor A; WES, whole-exome sequencing; WGBS, whole-genome bisulfite sequencing; WGS, whole-genome sequencing.
